# Reactions with Criegee intermediates are the dominant gas-phase sink for formyl fluoride in the atmosphere

**DOI:** 10.1016/j.fmre.2023.02.012

**Published:** 2023-03-07

**Authors:** Yu Xia, Bo Long, Ai Liu, Donald G. Truhlar

**Affiliations:** a College of Materials Science and Engineering, Guizhou Minzu University, Guiyang 550025, China; b Department of Chemistry, Chemical Theory Center, and Minnesota Supercomputing Institute, University of Minnesota, Minneapolis, MN 55455-0431, United States

**Keywords:** Atmospheric oxidation capacity, Criegee intermediates, Formyl fluoride, Reaction kinetics, CCSDT(Q)/CBS calculations

## Abstract

Atmospheric oxidation processes are of central importance in atmospheric climate models. It is often considered that volatile organic molecules are mainly removed by hydroxyl radical; however, the kinetics of some reactions of hydroxyl radical with volatile organic molecules are slow. Here we report rate constants for rapid reactions of formyl fluoride with Criegee intermediates. These rate constants are calculated by dual-level multistructural canonical variational transition state theory with small-curvature tunneling (DL-MS-CVT/SCT). The treatment contains beyond-CCSD(T) electronic structure calculations for transition state theory, and it employs validated density functional input for multistructural canonical variational transition state theory with small-curvature tunneling and for variable-reaction-coordinate variational transition state theory. We find that the M11-L density functional has higher accuracy than CCSD(T)/CBS for the HC(O)F + CH_2_OO and HC(O)*F* + *anti*-CH_3_CHOO reactions. We find significant negative temperature dependence in the ratios of the rate constants for HC(O)F + CH_2_OO/*anti*-CH_3_CHOO to the rate constant for HC(O)*F* + OH. We also find that different Criegee intermediates have different rate-determining-steps in their reactions with formyl fluoride, and we find that the dominant gas-phase removal mechanism for HC(O)F in the atmosphere is the reaction with CH_2_OO and/or *anti*-CH_3_CHOO Criegee intermediates.

## Graphical abstract

**Figure:**
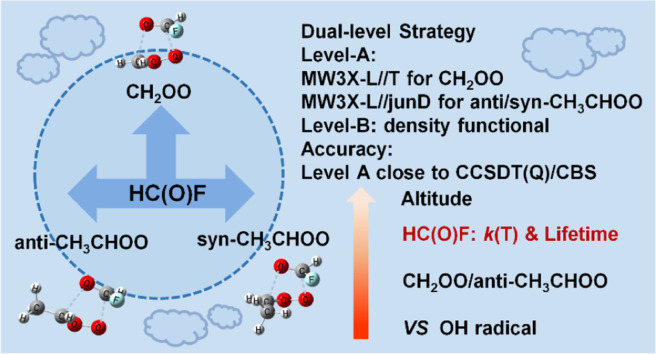

## 1 Introduction

Criegee Intermediates (CIs) are carbonyl oxides produced in the ozonolysis of alkenes [1,2], and they are of great interest in atmospheric modeling because of their important contributions to the production of oxidizing OH radicals [3], [4], [5], [6], [7], [8], [9], [10] and to the formation of secondary organic aerosols [11], [12], [13], [14], [15].

Criegee intermediates can act as dominant oxidizing sinks for various atmospheric molecules [8,[16], [17], [18]. The importance of the oxidative capacity of Criegee intermediate was firstly suggested by Cox and Penkett in their study of the rapid oxidation of sulfur dioxide (SO_2_) in the presence of ozone reactions with alkenes [19], [20], [21]. Recently, field measurements have also shown rapid reactions of sulfur dioxide with Creigee intermediates; these reactions make a large contribution to the formation of sulfuric acid [16], [17], [18], [19], [20], [21], [22]. Other experimental investigations have shown that other atmospheric acids such as formic acid, nitric acid, and hydrogen chloride [12,[23], [24], [25], [26], [27], [28], [29], [30], [31] can also be removed by Criegee intermediates because the reactions of Criegee intermediates with atmospheric acids are much faster than those of the corresponding reactions with OH. For example, the rate constant of the CH_2_OO + HCOOH reaction approaches the collision limit and makes a major contribution to the sink of formic acid in the gas phase of atmosphere [27].

Previous investigations indicated that aldehydes can react with Criegee intermediates; these processes make limited contributions to the degradation of unsubstituted aldehydes in the atmosphere because the reactions of aldehydes with carbonyl oxide cannot compete well with the aldehyde reactions with OH under atmospheric conditions [32], [33], [34], [35], [36].

**Fig. 1:**
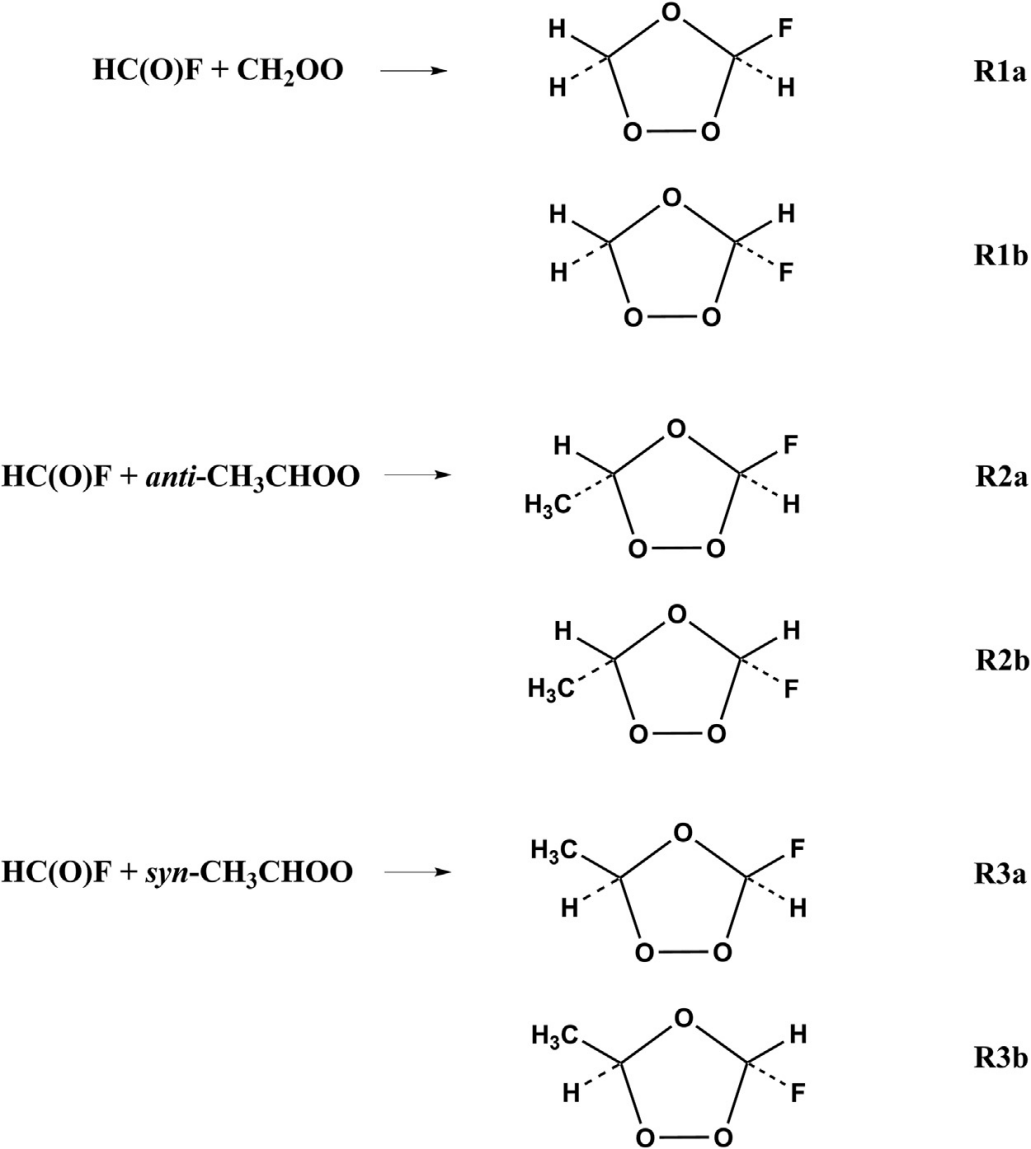
**Six reaction pathways considered inthis work.**

Formyl fluoride (which can be written either as HC(O)F or as FCHO) is an intermediate product in the atmospheric oxidation processes of hydrochlorofluorocarbons, hydrofluorocarbons, and hydrofluoro-olefins [37], [38], [39], [40], [41], [42], [43]. Although the atmospheric concentration of HC(O)F is unknown, HC(O)F is a main intermediate product in the atmospheric oxidation processes of (*E/Z*)−1,2,3,3,3-pentafluoropropene ((*E/Z*)-CF_3_CF=CHF) initiated by both Cl atom and OH radical[44], [45]. In this article, we report rate constants for the HC(O)F reactions with Criegee intermediates; in particular we examine the six reactions shown in Fig. 1.

Quantitative experimental study of the kinetics of Criegee intermediates by direct measurements is very difficult because they are reactive species generated by ozonolysis of unsaturated compounds, but theoretical calculations do not have the same limitations for studying elusive intermediates. The present theoretical study is concerned both with stabilized Criegee intermediates and with the dominant removal mechanism of HC(O)F due to gas-phase chemistry in the atmosphere; the reaction rates of nascent Criegee intermediates [46] are a separate issue. The present work has potential implications for the reactions of Criegee intermediates with other fluorinated aldehydes.

There are many uncertainties in atmospheric modeling. This paper uses previously validated methods to predict atmospheric chemistry for which there is no experimental data available, and this illustrates the ability of theoretical chemistry to contribute information to the pool of available data for atmospheric chemistry.

## 2 Theoretical methods and strategies

### 2.1 Dual-level method for kinetics

For the reactions of HC(O)F with CH_2_OO and *syn*-CH_3_CHOO, the rate constants are dominated at all temperatures by tight transition states. This is also true for HC(O)*F* + *anti*-CH_3_CHOO at high temperature, but at low temperature, this reaction rate is determined by the loose variational transition state for forming the precursor complex (which is the complex that is reached prior to the saddle point). Therefore, we considered both the loose and tight transition states for the HC(O)*F* + *anti*-CH_3_CHOO reaction.

Rate constants for all tight transition states were calculated by a dual-level (DL) strategy [47], [48], [49], [50],[56], [57], [58] that has been employed in several previous papers. The previous investigations have shown that the dual-level strategy can be used to obtain quantitative kinetics [48,[50], [51], [52],[56], [57], [58]. The dual-level strategy combines a conventional-transition-state-theory rate constant [53]$\;k_{\text{SS}-\text{TST}}^{\text{HL}}\;$based on a higher-level (HL) input with transmission coefficients calculated by direct-dynamics with a lower level (LL), in particular with a density functional validated specifically for the reaction under consideration. In previous work, we included a recrossing transmission coefficient $\Gamma_{\text{CVT}}^{\text{LL}}$ and a tunneling transmission coefficient ($\kappa_{SCT}^{LL})\;$calculated by reaction-path variational transition state theory, in particular by canonical variational theory with small-curvature [54,55] tunneling (CVT/SCT); this yields DL-CVT/SCT. Here we also add a multistructural (MS) transmission coefficient, $F_{\text{fwd}}^{\text{MS}-T}$; this yields DL-MS-CVT/SCT.

By following the procedure of the previous paragraph, the rate constants for the HC(O)F + CH_2_OO and HC(O)*F* + *syn*-CH_3_CHOO reactions are given by

(1) $$k_{\text{MS}-\text{CVT}/\text{SCT}}^{\text{DL}}=F_{\text{fwd}}^{\text{MS}-T}k_{\text{CVT}/\text{SCT}}^{\text{DL}}$$

where

(2) $$k_{\text{CVT}/\text{SCT}}^{\text{DL}}=\kappa_{SCT}^{LL}\;\Gamma_{\text{CVT}}^{\text{LL}}\;k_{\text{SS}-\text{TST}}^{\text{HL}}$$

and where all factors in Eqs. 1,2 are temperature dependent. Note that, although there are two transition states for the reaction studied here, the HL rate constant in Eq. 2 depends only on the lower-energy one. Because we are considering the high-pressure limit, tunneling is included at all energies above the zero-point energy of the precursor complex. More details are provided in Supplementary materials (SM).

Section 2.2 explains the benchmark-quality electronic-structure method, Section 2.3 explains the validated density functional method, and Section 2.4 explains the methods used for calculating the kinetics of HC(O)*F* + *anti*-CH_3_CHOO.

### 2.2 Electronic structure: higher-level methods

Reliable theoretical predictions require the most critical energetic input obtained by higher-level electronic structure theory. The inclusion of beyond-CCSD(T) contributions is important for CH_2_OO because it is strongly correlated [56]. For the HC(O)F + CH_2_OO reaction, benchmark electronic structure calculations were performed by using the MW3X-L//T composite method, in which we used MW3X-L for single-point calculations at geometries optimized by method T. The MW3X-L method [57,58] is a composite method that involves a combination of MW2-F12 [59] to approximate the CCSD(T)/CBS level (where CBS denotes complete basis set) and W3X-L [60] to obtain beyond-CCSD(T) contributions. The abbreviation “T” is our shorthand notation in this article for CCSD(T)-F12a [61,62]/cc-pVTZ-F12 [63]. Method T is also used for the higher-level frequency calculations.

We also studied the HC(O)F + CH_2_OO reaction by several other methods. This was done to test lower-cost methods that are affordable for the reactions of HC(O)F with *syn*- or *anti*-CH_3_CHOO. The highest-level methods we studied for this purpose and their shorthand abbreviations are as follows:

1. MW3X-L//junD: MW3X-L//CCSD(T)-F12a/jun-cc-pVDZ
2. MW3X-L//D: MW3X-L//CCSD(T)-F12a/cc-pVDZ-F12
3. W3X-L//T: W3X-L//CCSD(T)-F12a/cc-pVTZ-F12
4. W3X-L/junD: / W3X-L/DF-CCSD(T)-F12b/jun-cc-pVDZ
5. W3X-L/D: / W3X-L/CCSD(T)-F12a/cc-pVDZ-F12
6. MW2-F12//T: MW2-F12//CCSD(T)-F12a/cc-pVTZ-F12
7. MW2-F12//D: MW2-F12//CCSD(T)-F12a/cc-pVDZ-F12
8. W2X//T: W2X//CCSD(T)-F12a/cc-pVTZ-F12
9. W2X//$\text{ju}n^{\prime }$D: W2X//DF-CCSD(T)-F12b/$\text{ju}n^{\prime }$-cc-pVDZ
10. W2X//junD: W2X//DF-CCSD(T)-F12b/jun-cc-pVDZ
11. W2X//D: W2X//CCSD(T)-F12a/cc-pVDZ-F12

Our tests showed high accuracy for MW3X-L//junD, and therefore this method was used as a benchmark for reactions of HC(O)F with *syn*- and *anti*-CH_3_CHOO. All abbreviations are explained in detail in Table S1 (tables with a prefix “S” are provided in Supplementary materials).

### 2.3 Electronic structure: method for direct dynamics

The M11-L density functional [64] employs dual-range local exchange to provide broad accuracy for both weakly correlated and strongly correlated molecules, and the MG3S basis set [65] is a minimally augmented, valence-triple-zeta, multiply polarized basis set that has shown good accuracy at reasonable cost. Thus, the M11-L/MG3S method was chosen for the direct dynamics calculations for the reactions of HC(O)F with *syn*- and *anti*-CH_3_CHOO.

### 2.4 Kinetics of the HC(O)*F* + *anti*-CH_3_CHOO reaction

For HC(O)*F* + *anti*-CH_3_CHOO, we employed a high-pressure approximation to the steady-state approximation. This yields a result equivalent to the canonical unified statistical theory (CUS) [66], [67], [68] with the assumption that the flux through the complex is much larger than the flux through either transition state and with tunneling included down the zero-point level of the complex; this yields

(3) $$k_{\text{CUS}}\left(T\right)=\;\frac{k_{\text{tight}}k_{\text{loose}}}{k_{\text{tight}}+k_{\text{loose}}}$$

where *k*_tight_ is given by $k_{\text{MS}-\text{CVT}/\text{SCT}}^{\text{DL}}$ and *k*_loose_ is given by variable-reaction-coordinate variational transition state theory (VRC-VTST) [69], [70], [71] for the loose transition state between reactants and the precursor complex. More information is provided in Supplementary materials.

Scale factors [72] were applied to vibrational frequencies to improve calculated zero-point vibrational energies by correcting for anharmonicity and systematic errors in electronic structure. We used 0.984, 0.981, and 0.985 for method-T, method-junD, and M11-L/MG3S, respectively (See Table S2.)

### 2.5 Software

All the density functional calculations were performed using *Gaussian 16* [73] and *MN-GFM* [74]. The coupled cluster methods were performed using *Molpro 2019* [75] and *MRCC* [76,77]. Rate constants were calculated using *Polyrate 2017C* [78] and *Gaussrate 2017B* [79]*.*

## 3 Results and discussion

In the following, all enthalpies, enthalpies of reaction, and enthalpies of activation are values calculated at 0 K.

**Fig. 2:**
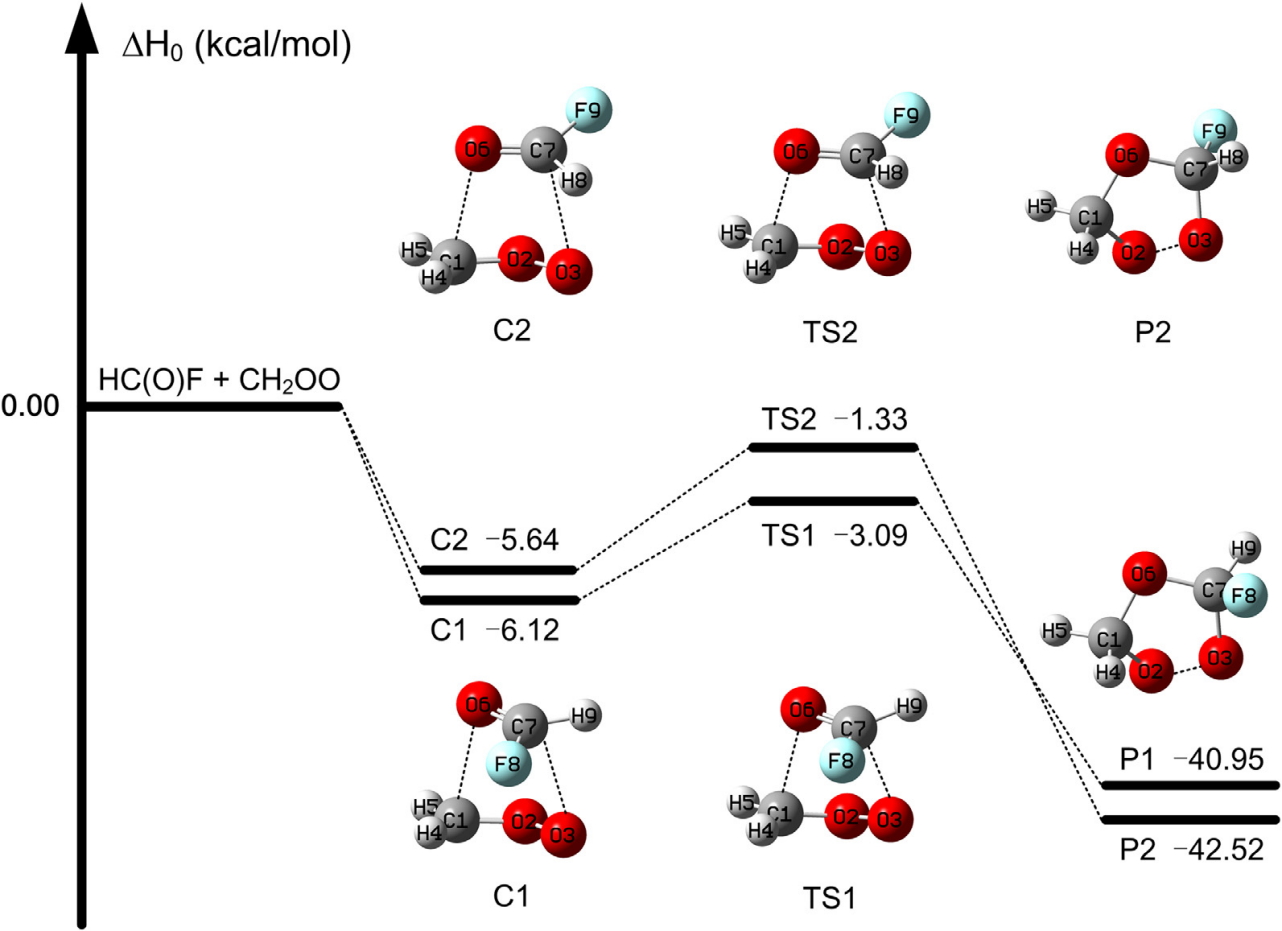
**Enthalpy profiles for the FCHO +CH**_**2**_**OO reaction calculated by MW3X-L//CCSD(T)-F12a/cc-pVTZ-F12 at 0 K.**

**Fig. 3:**
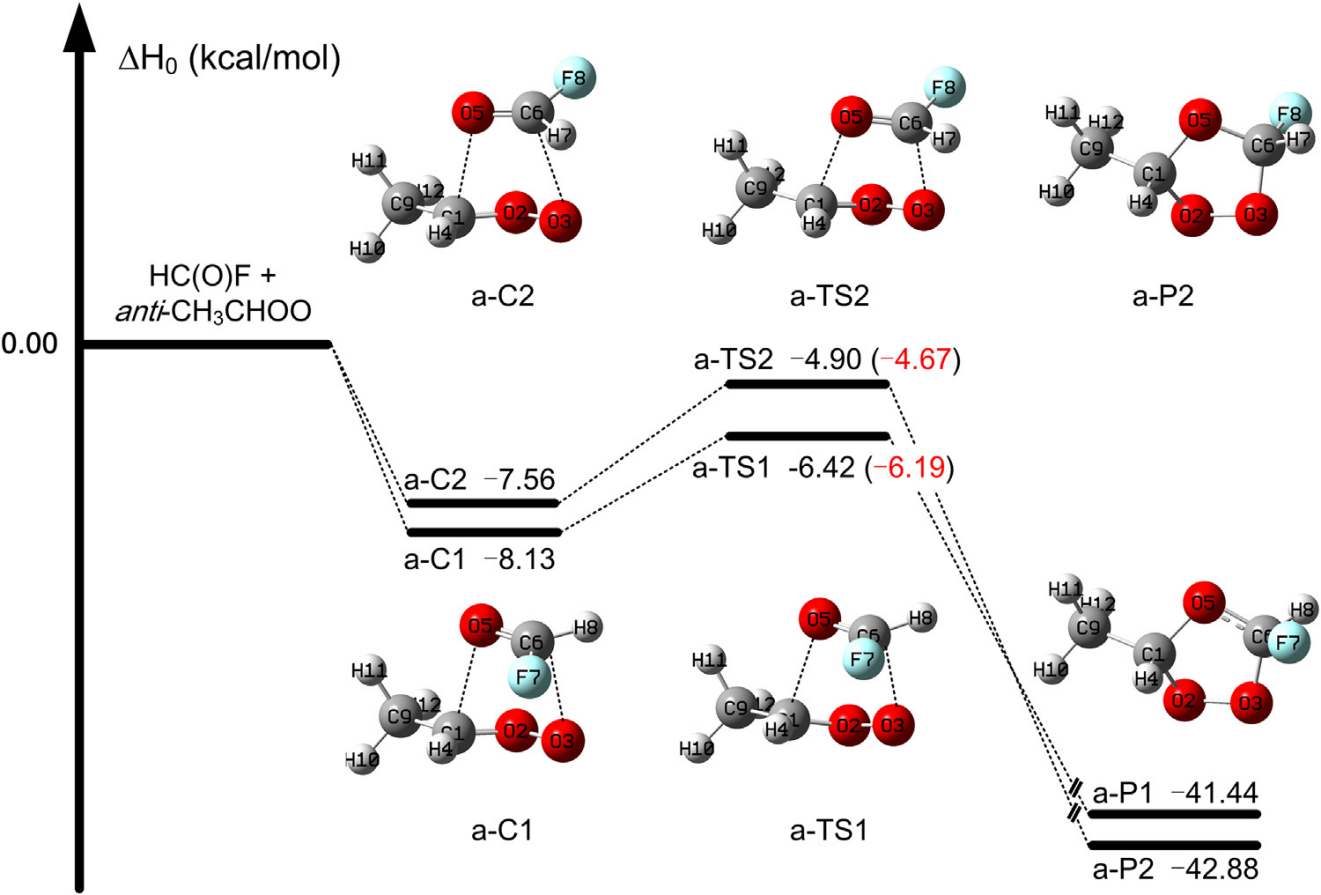
**The enthalpy profile at 0 K for the*****anti*****-CH**_**3**_**CHOO +FCHO reaction as calculated by W3X-L//junD level, and in parentheses, values are obtained for transition states at MW3X-L/junD level.**

**Fig. 4:**
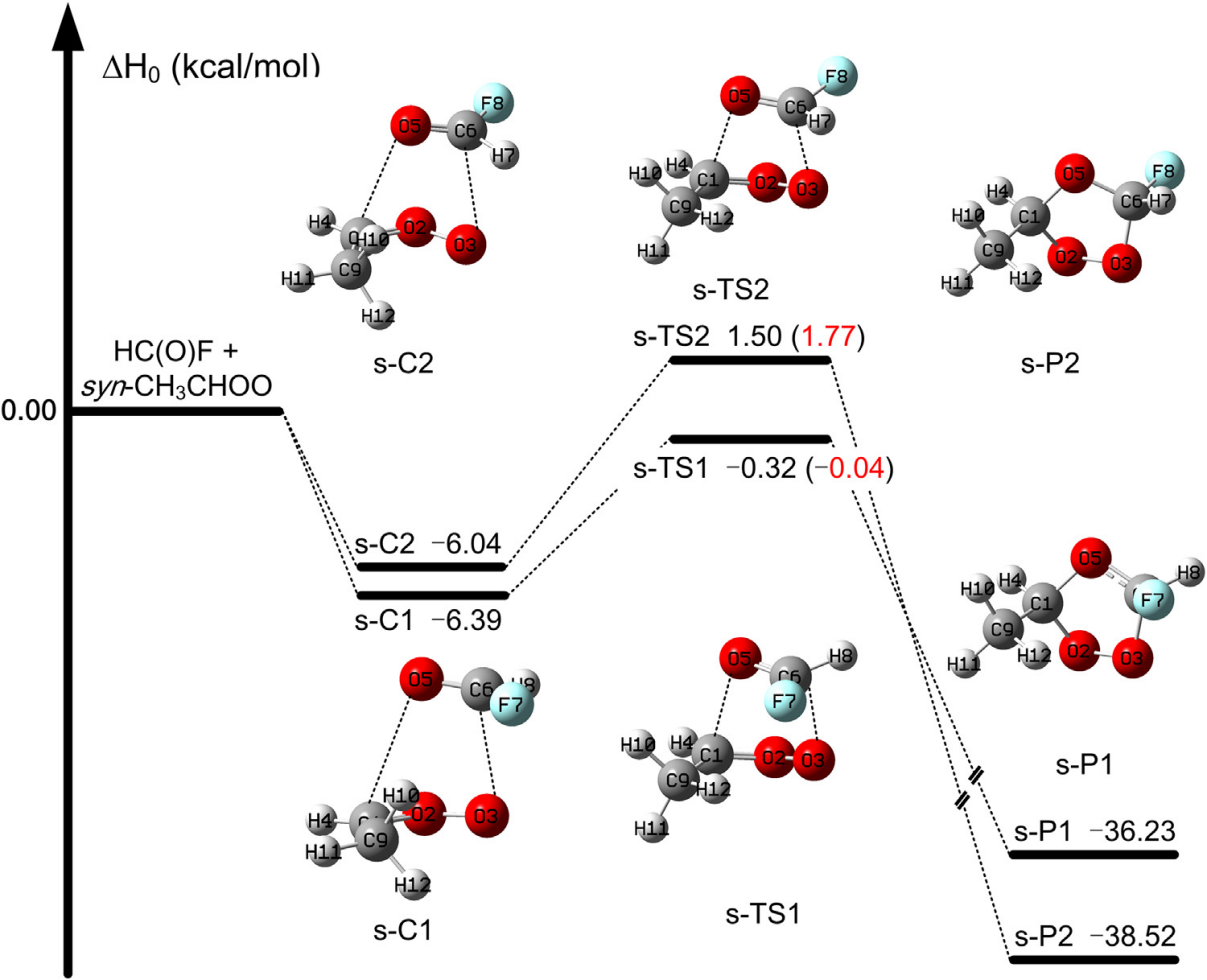
**The enthalpy profile at 0 K for the*****syn*****-CH**_**3**_**CHOO +FCHO reaction as calculated by W3X-L//junD level, and in parentheses, values are obtained for transition states at MW3X-L/junD level.**

The reactions under study proceed without a barrier to a precursor complex (with two conformers), pass through a tight transition state (with two conformers), and yield two products, as shown in Fig. 2, Fig. 3, Fig. 4. The figures also explain the labels (C1, a-TS2, s-P1, etc.) used for the various structures. These reactions are similar to HCHO + CH_2_OO [80,81] except for the stereoisomerism. There is an internal-rotation transition state connecting the conformers of the complex. We verified the nature of this transition state by examination of the direction of the imaginary-frequency normal mode; for the C1 and C2 conformers in the CH_2_OO case, this transition state has an enthalpy of activation of 1.5 kcal/mol with respect to C1 (as calculated by M11-L/MG3S; see Fig. S2).

Mean unsigned deviations (MUDs) are with respect to our best estimates, which (as explained above) are MW3X-L//T for CH_2_OO and MW3X-L//junD for the methyl-substituted cases.

### 3.1 Electronic structures of species in the HC(O)F + CH_2_OO reaction

In the HC(O)F + CH_2_OO reaction, the lower transition state is TS1 with an enthalpy of activation of –3.1 kcal/mol, which is 1.8 kcal/mol lower than that of TS2; this shows that this reaction mainly occurs via the transition state TS1. Fig. 2 shows that the reaction is exothermic with calculated enthalpies of reaction of −41.0 and −42.5 kcal/mol for the two products.

Tables S3, S4, and S5 compare results for the HC(O)F + CH_2_OO reaction as obtained with electronic structure levels 8–11 of Section 2.2. We see that these results agree with each other within 0.1 kcal/mol for the energy and enthalpy of complexation, for the barrier height, for the enthalpy of activation, and for the energy and enthalpy of reaction. Since these levels differ only in geometry, this indicates good agreement among the optimized structures.

Table 1 shows that the MUD of the enthalpies calculated by MW2-F12 (which is our best approximation to CCSD(T)/CBS) is 0.72 kcal/mol; this large deviation shows that the popular CCSD(T)/CBS level is insufficient for the CH_2_OO reaction with HC(O)F. The difference between the MW3X-L//T and MW2-F12 enthalpies for TS1 is 0.54 kcal/mol, which is close to the value of 0.54 kcal/mol that was obtained for the mean difference between CCSD(T)/CBS and post-CCSD(T) transition-state calculations in the CH_2_OO + NH_3_, H_2_S, and (H_2_O)_2_ reactions [50]. The difference of 0.54 kcal/mol leads to a Boltzmann factor of 2.5 at 298 K and 3.5 at 216.65 K (the lowest temperature in the troposphere according to the U. S. standard atmosphere). Thus, the present results show that post-CCSD(T) calculations are necessary for obtaining quantitative rate constants of the reactions of Criegee intermediates in the troposphere.

**Table 1:** **The relativeenthalpies of the CH**_**2**_**OO +HC(O)F reaction at 0 K.**

Methods	$\Delta H_{0}$						MUD*^g^*
	C1*^d^*	C2 *^d^*	TS1*^e^*	TS2 *^e^*	P1*^f^*	P2*^f^*	
MW3X-L//T^a^	−6.12	−5.64	−3.09	−1.33	−40.95	−42.52	0.00
MW3X-L//D*^b^*	−6.18	−5.88	−3.10	−1.35	−40.97	−42.53	0.06
MW3X-L//junD*^c^*	−6.25	−5.78	−3.04	−1.26	−41.07	−42.61	0.10
W3X-L//T^a^	−6.21	−5.73	−3.29	−1.53	−41.04	−42.60	0.13
W3X-L/junD*^c^*	−6.34	−5.87	−3.23	−1.46	−41.15	−42.71	0.19
W3X-L//D*^b^*	−6.31	−6.00	−3.25	−1.49	−41.09	−42.67	0.20
M11-L/MG3S	−6.39	−5.85	−3.89	−2.02	−41.36	−43.50	0.56
M06CR/MG3S	−6.81	−6.52	−3.03	−1.38	−39.78	−41.26	0.69
MW2-F12//T^a^	−6.37	−5.88	−3.63	−1.92	−42.3	−43.87	0.72
MW2-F12//D*^b^*	−6.44	−6.11	−3.64	−1.94	−42.33	−43.90	0.78
W2X//T^a^	−6.46	−5.97	−3.83	−2.12	−42.39	−43.95	0.85
W2X//D*^b^*	−6.56	−6.23	−3.80	−2.09	−42.45	−44.03	0.92

a Single-point energy calculation based on method-T geometries, where method T is CCSD(T)-F12a/cc-pVTZ-F12, and the corresponding frequencies are also calculated with method T. ^b^Single-point energy calculation based on method-D geometries, where method D is CCSD(T)-F12a/cc-pVDZ-F12, and the corresponding frequencies are also calculated with method D. ^c^Single-point energy calculation based on method-junD geometries, where method junD is DF-CCSD(T)-F12b/jun-cc-pVDZ, and the corresponding frequencies are also calculated with method junD. ^d^C1 and C2 are the precursor complexes between CH_2_OO and HC(O)F. ^e^TS1 and TS2 are the transition states of the CH_2_OO + HC(O)F reaction. ^f^P1 and P2 are the products formed in the CH_2_OO + HC(O)F reaction. ^g^MUD is the mean unsigned derivation from the best estimate in the top row. ($\Delta H_{0}\;$All in kcal/mol).

Table 1 shows that the mean unsigned derivation (MUD) of the M11-L/MG3S enthalpies from our best estimate by MW3X-L//T is 0.56 kcal/mol; this is better than the MUDs of the much more expensive W2X//T and MW2-F12//T. The calculated results show that the M11-L functional combined with MG3S basis set is reasonably reliable for describing the CH_2_OO + HC(O)F reaction. Thus, M11-L/MG3S has been used to do direct dynamics calculations.

Table 1 shows that MW3X-L//junD enthalpies have a mean unsigned deviation of only 0.1 kcal/mol from the MW3X-L//T enthalpies; this indicates that MW3X-L//junD method is reliable for electronic structure calculations in the CH_2_OO + HC(O)F reaction. Thus, we used the MW3X-L//junD method to do benchmark calculations for the *anti-* and *syn*-CH_3_CHOO reactions. We note that the junD basis can produce a larger deviation in other Criegee reactions [82].

Our best estimate of the enthalpy of activation (−3.1 kcal/mol) is 2.2 kcal/mol higher than the enthalpy of activation of HCHO + CH_2_OO (as calculated by W3X-L//CCSD(T)-F12a/cc-pVTZ-F12 [50]; this shows that the reactivity of HC(O)F is weakened by the fluorine substituent. This tendency is also shown in the comparison of OH and HO_2_ reactions with HC(O)F [83,99], and OH and HO_2_ reactions with HCHO [84,85]. However, comparison of Figs. 2 and S1 shows that our best estimate of the enthalpy of activation of CH_2_OO + HC(O)F is 6.6 kcal/mol lower than that of HC(O)*F* +OH (estimated by W3X-L//CCSD(T)-F12b/jun-cc-pVDZ); this indicates that the HC(O)F + CH_2_OO reaction is faster than HC(O)*F* + OH.

### 3.2 Electronic structures of species in the reactions of HC(O)F with *anti*- and *syn-*CH_3_CHOO

In the HC(O)*F* + *anti*-CH_3_CHOO reaction, the lower transition state is a-TS1 with an enthalpy of activation is −6.19 kcal/mol calculated by MW3X-L//junD; this is 1.52 kcal/mol lower than that of a-TS2, as listed in Table S6. This shows that this reaction occurs more readily via the transition state a-TS1 than via the transition state a-TS2. The enthalpy of activation for a-TS1 in Table S6 is −6.19 kcal/mol (as calculated by MW3X-L/junD), which is 3.1 kcal/mol lower than that of TS1 in Table 1 (as calculated by MW3X-L/T); this indicates that the introduction of the CH_3_ group in the *anti* site of CH_2_OO decreases the barrier height of the reaction with HC(O)F. The HC(O)*F* + *anti*-CH_3_CHOO reaction is exothermic with calculated enthalpies of reaction of −41.4 and −42.9 kcal/mol, as shown in Fig. 3.

For the HC(O)*F* + *syn*-CH_3_CHOO reaction, our best estimate is that the transition state s-TS1 has an enthalpy of activation of −0.04 kcal/mol, which is 1.8 kcal/mol lower than that of transition state s-TS2, as listed in Table S6 and Fig. 4. However, the enthalpy of activation is −0.04 kcal/mol for s-TS1 at the MW3X-L/junD level, which is 6.2 and 3.1 kcal/mol higher than those of a-TS1 and TS1, respectively. This shows that the introduction of a CH_3_ group with *syn* stereochemistry increases the barrier height of the HC(O)F reaction with Criegee intermediates. The HC(O)*F* + *syn*-CH_3_CHOO reaction is exothermic with calculated enthalpies of reaction of −36.2 and −38.5 kcal/mol in Fig. 4.

### 3.3 Kinetics of HC(O)F + CH_2_OO, *anti*-CH_3_CHOO, and *syn*-CH_3_CHOO

We computed the rate constants in the temperature range from 190 to 350 K, and they are given in Table 2. Intermediate steps of the rate constant calculations are reported in Tables S7-S9.

**Table 2:** **The calculatedrate constants and the corresponding Arrhenius activation energies at 190–350 K.**

T (K)	*k*_1_^a^	*k*_2_*^b^*	*k*_3_*^c^*	*E*_a1_^a^	*E*_a2_*^b^*	*E*_a3_*^c^*
190	4.67 × 10^−12^	7.82 × 10^−10^	5.61 × 10^−16^	−3.71	−0.30	−0.51
200	2.95 × 10^−12^	6.76 × 10^−10^	5.25 × 10^−16^	−3.58	−1.53	−0.48
210	1.96 × 10^−12^	5.41 × 10^−10^	4.96 × 10^−16^	−3.47	−2.56	−0.46
220	1.35 × 10^−12^	3.97 × 10^−10^	4.72 × 10^−16^	−3.38	−3.42	−0.44
230	9.69 × 10^−13^	2.71 × 10^−10^	4.53 × 10^−16^	−3.30	−4.13	−0.41
240	7.18 × 10^−13^	1.79 × 10^−10^	4.37 × 10^−16^	−3.24	−4.70	−0.38
250	5.46 × 10^−13^	1.17 × 10^−10^	4.23 × 10^−16^	−3.18	−5.14	−0.36
260	4.25 × 10^−13^	7.72 × 10^−11^	4.12 × 10^−16^	−3.14	−5.48	−0.33
270	3.39 × 10^−13^	5.16 × 10^−11^	4.03 × 10^−16^	−3.10	−5.72	−0.30
280	2.75 × 10^−13^	3.53 × 10^−11^	3.95 × 10^−16^	−3.08	−5.88	−0.28
290	2.27 × 10^−13^	2.46 × 10^−11^	3.89 × 10^−16^	−3.06	−5.96	−0.25
**298**	**1.97×10^−13^**	**1.88×10^−11^**	**3.85×10^−16^**	**−3.05**	**−5.97**	**−0.23**
300	1.90 × 10^−13^	1.76 × 10^−11^	3.84 × 10^−16^	−3.05	−5.97	−0.22
310	1.62 × 10^−13^	1.28 × 10^−11^	3.79 × 10^−16^	−3.04	−5.91	−0.19
320	1.39 × 10^−13^	9.57 × 10^−12^	3.76 × 10^−16^	−3.04	−5.80	−0.16
330	1.21 × 10^−13^	7.26 × 10^−12^	3.73 × 10^−16^	−3.05	−5.64	−0.13
340	1.07 × 10^−13^	5.60 × 10^−12^	3.71 × 10^−16^	−3.06	−5.42	−0.11
350	9.49 × 10^−14^	4.40 × 10^−12^	3.70 × 10^−16^	−3.07	−5.17	−0.08

a k_1_ and E_a1_ are the rate constants and Arrhenius activation energies of the HC(O)F + CH_2_OO reaction. ^b^k_2_ and E_a2_ are the rate constants and Arrhenius activation energies of the HC(O)*F* + anti-CH_3_CHOO reaction. ^c^k_3_ and E_a3_ are the rate constants and Arrhenius activation energies of the HC(O)*F* + syn-CH_3_CHOO reaction. (k_1_, k_2_, and k_3_ in cm^3^ molecule^−1^ s^−1^; E_a1_, E_a2_, and E_a3_ in kcal/mol).

In the present work, we only consider the high-pressure limiting rate constants (i.e.; rate constants corresponding to thermally equilibrated vibrational-rotational states of the reactants) for the reactions of CH_2_OO, *anti-*CH_3_CHOO, and *syn*-CH_3_CHOO with formyl fluoride because our previous investigations have shown that the rate constant for the similar CH_2_OO + HCHO reaction is independent of pressure [50]. The calculated rate constants are fitted using the following four-parameter [86], [87], [88] function in the temperature range from 190 to 350 K:

(4) $$k=A{\left(\frac{T+T_{0}}{300}\right)}^{n}\text{exp}\left[-\frac{E\left(T+\;T_{0}\right)}{R\left(T^{2}+\;T_{0}^{2}\right)}\right]$$

where *R* is the gas constant, and *T* is temperature in K. The fitting parameters are listed in Table S10.

The Arrhenius activation energies are also obtained as functions of temperature by using

(5) $$E_{a}=-R\frac{d\ln k}{d\;\left(1/T\right)}$$

**Fig. 5:**
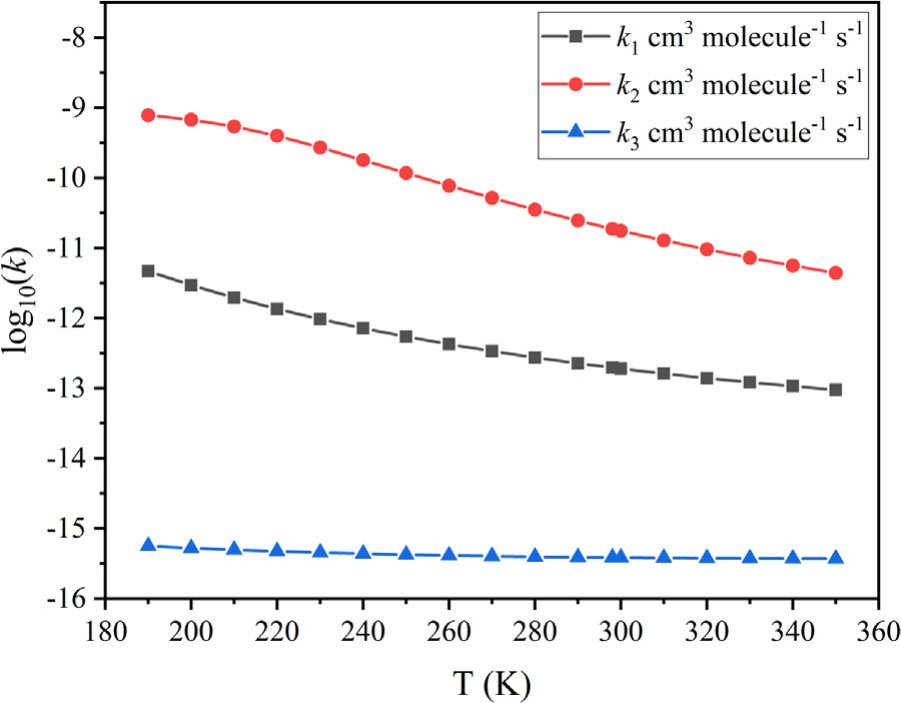
**The high-pressure-limit rateconstants of the reaction of FCHO with CH**_**2**_**OO/*****anti*****-CH**_**3**_**CHOO/*****syn*****-CH**_**3**_**CHOO at the temperature range of 190–350 K.**

**Fig. 6:**
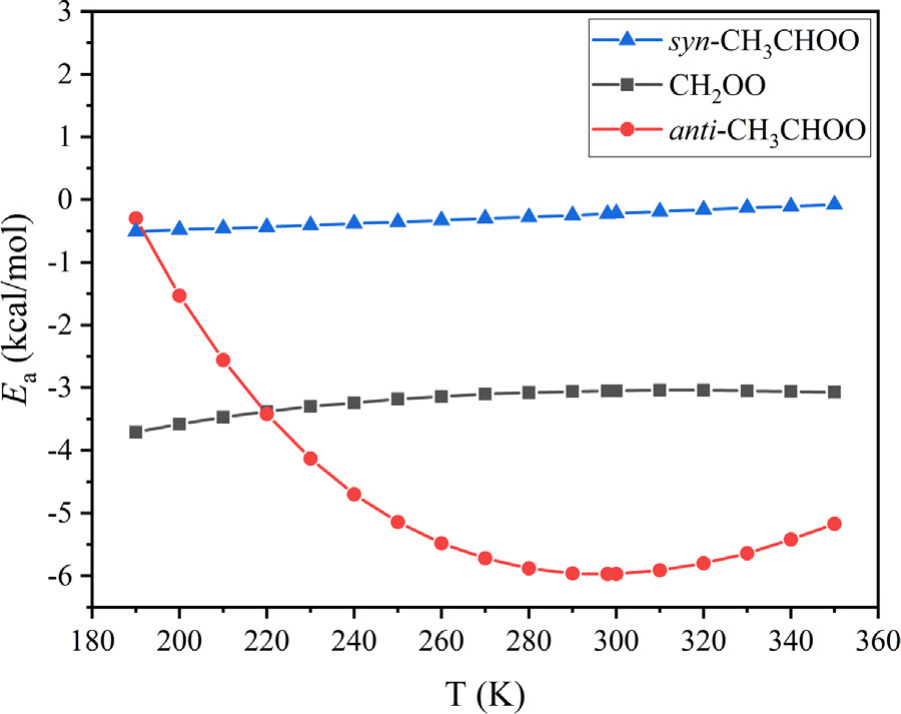
**Arrheniusactivation energies of the reaction of FCHO with CH**_**2**_**OO/*****anti*****-CH**_**3**_**CHOO/*****syn*****-CH**_**3**_**CHOO at the temperature range of 190 – 350 K.**

We find that the rate constants of CH_2_OO + HC(O)F have a negative temperature dependence, as shown in Table 2 and Fig. 5, and the calculated activation energies increase from –3.71 to –3.07 kcal/mol as the temperature increases from 190 to 350 K, as plotted in Fig. 6.

The rate constant *k*_2_ of the HC(O)*F* + *anti*-CH_3_CHOO reaction decreases from 7.82 × 10^−10^ cm^3^ molecule^−1^ s^−1^ at 190 K to 4.40 × 10^−12^ cm^3^ molecule^−1^ s^−1^ at 350 K. The rate constant *k*_3_ of the HC(O)*F* + *syn*-CH_3_CHOO reaction also decreases with the increase of temperature, but not nearly as rapidly for temperatures above 200 K, and the calculated activation energies decrease from −0.08 to −0.51 kcal/mol between 190 and 350 K.

### 3.4 Atmospheric implications

In gas-phase atmospheric chemistry, HC(O)F can undergo photolysis, hydrolysis, and reaction with HO and HO_2_. However, previous investigations have concluded that the main sink of HC(O)F in the atmosphere is reaction with OH [43,[89], [90], [91],99]. Here, we re-examine this conclusion by considering the atmospheric depletion of HC(O)F by CH_2_OO, *anti*-CH_3_CHOO, *syn*-CH_3_CHOO, and OH. The ratios of the removal rates by the reactions with Criegee intermediates to the removal rate by the reaction with OH are as follows:

(6) $$R_{C/OH}=\frac{k_{1}\left[CH_{2}\text{OO}\right]}{k_{\text{OH}}\;\left[\text{OH}\right]}$$

(7) $$R_{a/OH}=\frac{k_{2}\left[anti-CH_{3}\text{CHOO}\right]}{k_{\text{OH}}\;\left[\text{OH}\right]}$$

(8) $$R_{s/OH}=\;\frac{k_{3}\left[syn-CH_{3}\text{CHOO}\right]}{k_{\text{OH}}\;\left[\text{OH}\right]}$$

where *k*_1_, *k*_2_, and *k*_3_ are the bimolecular rate constants for HC(O)F reacting with CH_2_OO, *anti*-CH_3_CHOO, and *syn*-CH_3_CHOO, respectively, and $k_{\text{OH}}$ is the bimolecular rate constant of HC(O)*F* + OH. For use in these equations, $k_{\text{OH}}$ has been calculated by using our dual-level strategy; detailss are provided in Supplementary materials.

The values of *R*_C/OH_, *R*_a/OH_, and *R*_s/OH_ depend not only on the rate constants, but also on the concentrations of the four competitive reactants. The literature estimates of the concentrations of Criegee intermediates are in the range between 10^4^ and 10^5^ molecules/cm^3^ [92,93], and those of OH are in the range 10^4^ to 10^6^ molecules/cm^3^ during the daytime [94], [95], [96], [97], The calculated rate ratios of Eq. 3 are given in Table S11. The calculated results show that the CH_2_OO + HC(O)F reaction dominates HC(O)*F* + OH in the temperature range between 190 and 300 (see Table S11) even when the concentrations of OH and CH_2_OO are 10^6^ and 10^4^ molecules/cm^3^, respectively. The concentration of CH_2_OO varies from one area to another area, and there is no data for the altitude dependence. Thus, the rate ratio has some uncertainty.

The rate ratio of CH_2_OO + HC(O)F to HC(O)*F* + OH has a large negative temperature dependence; such a negative temperature dependence is expected to also apply to reactions of Criegee intermediates with other aldehydes and ketones.

Table S12 compares the atmospheric lifetimes of HC(O)F with respect to reaction with CH_2_OO, OH, and HO_2_ at 220–320 K. We set [HO_2_] = 1.1 × 10^9^ molecules/cm^3^, which is close to a recent measurement [98] of the average daytime concentration. (The nighttime concentration is considerably lower.) This comparison shows that the reaction with CH_2_OO dominates for temperatures of 290 K and below, even when we assume this high [HO_2_] concentration and a low [CH_2_OO] concentration. This changes the current thinking about the dominant sink for HC(O)F in the atmosphere.

Table 3 provides the rate ratios *R*_C/OH_, *R*_a/OH_, and *R*_s/OH_ as functions of altitude at 0–15 km. For this table, we assume that the concentrations of CH_2_OO and CH_3_CHOO are 10^4^ molecules/cm^3^, which are taken here to be independent of altitude because the vertical distribution of Criegee intermediates is unknown. The ratio *R*_C/OH_ increases from 5.83 at 5 km to 344.81 at 15 km, showing the CH_2_OO + HC(O)F reaction dominates the OH + HC(O)F reaction at 5 – 15 km. The ratio *R*_a/OH_ increases from 51.5 at 0 km to 9930 at 15 km, and this indicates that the *anti*-CH_3_CHOO + HC(O)F reaction dominates the OH + HC(O)F reaction over the 0–15 km range. However, for the assumed concentrations, the ratio *R*_s/OH_ is less than one over the 0–15 km range.

**Table 3:** **The rateconstants and rate ratios as functions of altitude in the troposphere.**

H^a^ (km)	T^a^ (K)	*k*_1_*^b^*	*k*_2_*^b^*	*k*_3_*^b^*	*k*_OH_*^c^*	[OH]*^d^*	R_C/H_*^e^*	R_a/H_*^f^*	R_s/H_*^f^*
0	290.2	1.75 × 10^−13^	2.45 × 10^−11^	3.89 × 10^−16^	1.59 × 10^−15^	3.0 × 10^6^	0.37	51.5	0.00
5	250.5	4.07 × 10^−13^	1.15 × 10^−10^	4.23 × 10^−16^	6.98 × 10^−15^	1.0 × 10^6^	5.83	1.65 × 10^3^	0.01
10	215.6	1.18 × 10^−12^	4.58 × 10^−10^	4.83 × 10^−16^	2.87 × 10^−15^	5.7 × 10^5^	72.4	2.80 × 10^4^	0.03
15	198	2.44 × 10^−12^	7.03 × 10^−10^	5.32 × 10^−16^	1.69 × 10^−16^	4.2 × 10^5^	344	9.93 × 10^4^	0.08

a Temperature as a function of altitude are from Brasseur, G.; Solomon, S. Aeronomy of the Middle Atmosphere: Chemistry and Physics of the Stratosphere and Mesosphere, Springer Netherlands: Dordrecht, 1986; pp. 441–441. ^b^k_1_, k_2_, and k_3_ (in cm^3^ molecule^−1^ s^−1^) are the rate constants of the HC(O)F reactions with CH_2_OO, anti-CH_3_CHOO, and syn-CH_3_CHOO, respectively. ^c^k_OH_ (in cm^3^ molecule^−1^ s^−1^) is the rate constant of the HC(O)F reaction with OH. ^d^The concentration (in molecules/cm^3^) of OH at 5–15 km is obtained from Brasseur, G.; Solomon, S.; Aeronomy of the Middle Atmosphere: Chemistry and Physics of the Stratosphere and Mesosphere, the third revised and enlarged edition, Springer Netherlands: Dordrecht, 2005. The concentration of OH at 0 km is obtained from the literature [97]. ^e^The rate ratio R_C/H_$=\frac{k_{1}\left[CH_{2}\text{OO}\right]}{k_{\text{OH}}\left[\text{OH}\right]}$, where [CH_2_OO] is assumed to be 10^4^ molecules/cm^3^ at all altitudes. ^f^The rate ratios R_a/H_$=\frac{k_{2}\left[\text{sCI}\right]}{k_{\text{OH}}\left[\text{OH}\right]}$ and R_s/H_$=\frac{k_{3}=\left[\text{sCI}\right]}{k_{\text{OH}}\left[\text{OH}\right]}$, where [sCI] is the concentration of stabilized Criegee intermediate that is assumed to be 10^4^ molecules/cm^3^ at all altitudes.

## 4 Conclusion

The MW3X-L method, which includes beyond-CCSD(T) contributions, was used with CCSD(T) geometries and vibrational frequencies to calculate transition state theory rate constants for the reactions of HC(O)F with CH_2_OO, *anti*-CH_3_CHO, and *syn*- CH_3_CHO. Transmission coefficients to account for recrossing, tunneling, and multistructural effects were added with a validated density functional method (M11-L/MG3S), and high-pressure-limit rate constants were calculated by dual-level multistructural canonical variational transition state theory with small-curvature tunneling. We find that beyond-CCSD(T) contributions are necessary for obtaining quantitative barrier heights. We also find that the accuracy of M11-L/MG3S is higher than that of CCSD(T)/CBS for the CH_2_OO + HC(O)F reaction. The present investigations show that the reactions of HC(O)F with CH_2_OO and *anti*-CH_3_CHOO occur about two orders of magnitude faster than the reaction of HC(O)F with OH. We find that CH_2_OO and *anti*-CH_3_CHOO can be the dominant sink of HC(O)F in the atmosphere, and therefore they upset the accepted view that HC(O)F is mainly removed by hydroxyl radical [43,99]. They also provide new insight into atmospheric oxidation capacity, and in this respect, they have broad implications in atmospheric chemistry. The present findings directly elucidate the atmospheric lifetimes of formyl fluoride, and they are also relevant to atmospheric modeling of hydrochlorofluorocarbons (HCFCs), hydrofluorocarbons (HCFs), and hydrofluoro-olefins (HFOs) because very recently, HC(O)*F* + OH was still considered to be the main sink for HC(O)F in modeling degradation of hydrofluoro-olefins (HFOs) [43].

## Declaration of competing interest

The authors declare that they have no conflicts of interest in this work.

## Appendix Supplementary materials

Image, application 1
