# Supplementary material for: Reactions with Criegee intermediates are the dominant gas-phase sink for formyl fluoride in the atmosphere
Source: Fundam Res. 2023 Mar 7;4(5):1216–24. doi: 10.1016/j.fmre.2023.02.012 (PMC11489503; doi:10.1016/j.fmre.2023.02.012)
Supplement: Supplementary file 1 [file mmc1.docx]

Supplementary materials

February 21, 2023

**Reactions with Criegee Intermediates Are the Dominant Gas-Phase Sink
for** **Formyl Fluoride in the Atmosphere**

Yu Xia,*^a^* Bo Long,*^a^*^[[1]](#footnote-1)^* Ai Liu,*^a^* Donald G. Truhlar*^b^**

*^a^*College of Materials Science and Engineering, Guizhou Minzu University, Guiyang 550025, China

*^b^*Department of Chemistry, Chemical Theory Center, and Supercomputing Institute, University of Minnesota, Minneapolis, Minnesota 55455-0431, USA

**Table of Contents**

Note on units S-3

Software S-3

Electronic partition functions S-3

Computational details of the DL-MS-CVT/SCT calculations S-4

Details of the variable-reaction-coordinate rate calculations for the
*anti*-CH_3_CHOO + FCHO reaction S-5

Computational details for the FCHO + OH reaction S-5

Table S1. Abbreviations for the theoretical methods and basis sets S-6

Table S2. The scale factors for vibrational frequencies S-7

**CH_2_OO reaction S-7**

Table S3. Mean unsigned deviations of complexation energies, barrier heights,
and reaction energies for the CH_2_OO + FCHO reaction at 0 K S-7

Table S4. Mean unsigned deviations of relative zero-point vibrational energies
for the CH_2_OO + FCHO reaction at 0 K S-7

Table S5. The enthalpies of activation and reaction enthalpies for the

CH_2_OO + FCHO reaction at 0 K S-8

**CH_2_OO, *anti*-CH_3_CHOO, and *syn*-CH_3_CHOO reactions S-8**

Table S6. The enthalpies of activation at 0 K for the reactions of FCHO with CH_2_OO,

*anti*-CH_3_CHOO, and *syn*-CH_3_CHOO S-8

Table S7. HPL rate constants and transmission coefficients for the

FCHO + CH_2_OO reaction S-9

Table S8. HPL rate constants and transmission coefficients for the

*anti*-CH_3_CHOO + FCHO reaction S-10

Table S9. HPL rate constants and transmission coefficients for the

*syn*-CH_3_CHOO + FCHO reaction S-11

Table S10. Fitting parameters of high-pressure-limit rate constants for forward reactions S-12

**CH_2_OO, OH, and HO_2_ reactions S-13**

Table S11. The ratio between CH_2_OO + FCHO and FCHO + OH reaction rates
for various concentrations S-13

Table S12. Atmospheric lifetimes of FCHO for reaction with CH_2_OO, OH, and

HO_2_ as functions of temperature S-14

**Geometries and absolute energies S-15**

Table S13. Cartesian coordinates (Å) of optimized structures S-15

Table S14. Absolute energies in hartrees S-25

**Figures S-27**

Figure S1. Enthalpy profiles at 0 K for the FCHO + OH reaction S-27

Figure S2. Enthalpy profile at 0 K for the conversion of C1 and C2 S-28

**References** **S-29**

**Note on units**

In the article and SI, all the energies and enthalpies are in kcal/mol (except for Table S16 which uses hartrees), all the frequencies are in cm^-1^, all coordinates are in Å and deg, all concentrations are in molecules/cm^3^, and all rate constants are in cm^3^·molecule^-1^·s^-1^.

**Software**

All the electronic structure calculations were performed using the *Gaussian 16*^[1]^ and *MN-GFM*^[2]^ software packages. The coupled cluster methods were performed by *Molpro 2019*^[3]^ and *MRCC*.^[4-5]^ Rate constants were calculated using *Polyrate* *2017B*^[6]^ and *Gaussrate* *2017B*.^[7]^

**Electronic partition functions**

The rate constants include the ratio of electronic partition functions

$\frac{ｇ_{\mathrm{TS}}}{ｇ_{\mathrm{FCHO}}ｇ_{OH or Criegee intermediate}}$.

where TS denotes the transition state.

For the purpose of computing electronic partition functions, we used 0.4 kcal/mol for the energy difference of the two spin-orbit-split states of OH(^2^Π). For the other species, we take the electronic partition function to be the electronic degeneracy of the ground state. These treatments yield:

*g*_TS_ = 2 for the reaction with OH and 1 for reactions of Criegee intermediates

*g*_FCHO_ = 1

*g*_OH_ ≈ 2 + 2exp[–(0.4 kcal/mol)/*RT*]

*g*_Criegee intermediate_ = 1

**Computational details of the DL-MS-CVT/SCT calculations**

The DL-MS-CVT rate constant is

$k_{MS-CVT/SCT}^{\mathrm{DL}}$(*T*) = $F_{\mathrm{fwd}}^{MS-T}k_{SS-CVT/SCT}^{\mathrm{DL}}$(*T*) (1)

where the single-structural CVT/SCT rate constant is defined as

$k_{SS-CVT/SCT}^{\mathrm{DL}}$(*T*) = ${\kappa_{SCT}^{LL}\Gamma}_{\mathrm{CVT}}^{\mathrm{LL}}k_{SS-TST}^{\mathrm{HL}}$(*T*) (2)

in which $\kappa_{SCT}^{LL}$ and $\Gamma_{\mathrm{CVT}}^{\mathrm{LL}}$ are the tunneling and recrossing transmission coefficients calculated by using canonical vibrational transition theory with small-curvature at the lower level (LL), and $k_{SS-TST}^{\mathrm{HL}}$ is the rate constant obtained by using conventional transition state theory at the higher level (HL). The multistructural and torsional anharmonicity factor^[8]^ of the forward reaction is defined as

$F_{\mathrm{fwd}}^{MS-T}= \frac{F^{MS-T}(\mathrm{TS})}{F^{MS-T}(R)}= \frac{Q^{\ddagger-MS-T}/Q^{\ddagger-SSHO}}{Q^{R-MS-T}/Q^{R-SSHO}}$ (S3)

in which $Q^{\ddagger-MS-T}$ and $Q^{R-MS-T}$are the multistructural partition functions for the saddle points and the reactants, respectively, and $Q^{\ddagger-SSHO}$ and $Q^{R-SSHO}$ are the single-structural harmonic-oscillator rovibrational partition functions for the lowest-energy saddle point and the reactant, respectively.

**Details of the variable-reaction-coordinate rate calculations for the** ***anti*-CH_3_CHOO + FCHO reaction**

In eq 3 of the main text, *k*_loose_ denotes the rate constant of the formation of the precursor complex from the reactants. This was calculated by using variable-reaction-coordinate variational transition state theory (VRC-VTST).^[9-11]^ We used two pivot points to produce a single-faceted dividing surface. One pivot point is located at a distance *d* from the center of mass (COM) of *anti*-CH_3_CHOO, where the vector connecting the pivot point with *anti*-CH_3_CHOO’s COM is perpendicular to the *anti*-CH_3_CHOO plane, and the other pivot point is located at a distance *d* from the COM of FCHO, where the vector connecting the pivot point with FCHO’s COM is perpendicular to FCHO plane. The lengths of these vectors were fixed at 0.05 Å because our investigations showed that the best results for the CH_2_OO + HCHO/(H_2_O)_2_ reaction were obtained with *d* = 0.05 Å.^[12]^

The reaction coordinate *s* is the distance between a pivot point on one reactant and a pivot point on the other reactant. The distance *s* between pivot points was varied from 4.3 to 8.4 Å with a 0.1 Å grid increment to find the optimum value. We used 1400 configurations for sampling the single-faceted dividing surfaces.

**Computational details for the FCHO + OH reaction**

The energies calculated for the FCHO + OH reaction are in Figure A1. The rate constant was calculated by the dual-level strategy^[13-18]^ that uses W3X-L^[19]^ //CCSD(T)-F12a^[20]^/jun-cc-pVDZ^[21]^ (called W3X-L//junD for short) as the higher-level theory for conventional transition state theory without tunneling and that uses M08-HX/MG3S^[22-23]^ as the lower-level theory for direct dynamics calculations of the recrossing transmission coefficient and the tunneling transmission coefficient by using canonical variational transition state theory with small curvature tunneling. The calculations for this reaction are single-structural.

Table S1. Abbreviations for the theoretical methods and basis sets used in the article*^a^*

| **Method** | **Ref.** | | **Explanation** |
| --- | --- | --- | --- |
| Electronic structure methods | | | |
| M11-L | [24] | Minnesota 2011 local density functional | |
| CCSD(T) | [25] | Coupled cluster theory with single and double excitations and noniterative connected triple excitations | |
| CCSD(T)-F12a | [20] | CCSD(T) with F12a explicit correlation | |
| CCSDT(Q) | [26] | Coupled cluster theory with single, double, and triple excitations and noniterative connected quadruple excitations | |
| W2X | [27] | Cost-effective approximation to CCSD(T)/CBS | |
| MW2-F12 | [28] | Cost-effective approximation to CCSD(T)/CBS with larger basis sets | |
| W3X-L | [19] | Cost-effective approximation to CCSDT(Q)/CBS with the larger basis sets | |
| MW3X-L | [15] | MW2-F12 plus beyond-CCSD(T) contributions from W3X-L | |
| Basis sets |  |  | |
| MG3S | [23] | Modified G3 semidiffuse basis, which is the same as  6-311+G (2df, 2p) for H, C, O, and F (although not for all elements) | |
| cc-pVTZ-F12 | [29] | correlation-consistent polarized valence double zeta basis modified for use in F12 calculations | |
| cc-pVDZ-F12 | [29] | correlation-consistent polarized valence double zeta basis modified for use in F12 calculations | |
| $\mathrm{jun}^{'}$-cc-pVDZ | new | Tables S1, S2, and S3 include some calculations labeled CCSD(T)-F12a/$\mathrm{jun}^{'}$-cc-pVDZ. These represent an improved way to carry out CCSD(T)-F12b calculations with the jun-cc-pVDZ basis set.  The original CCSD(T)-F12b/jun-cc-pVTZ calculations were performed using “cc-pVTZ/JKfit”, “cc-VTZ/MP2fit”, and “cc-pVTZ/OptR1” as auxiliary basis sets.  We define CCSD(T)-F12b/$\mathrm{jun}^{'}$-cc-pVTZ as a calculation using “aug-cc-pVDZ/JKfit”, “aug-cc-pVDZ/MP2fit”, and “aug-cc-pVDZ/OptR1” as auxiliary basis sets.  The cost of CCSD(T)-F12b/$\mathrm{jun}^{'}$-cc-pVDZ is almost equal to that of CCSD(T)-F12a/jun-cc-pVDZ. | |
| jun-cc-pVDZ | [21] | correlation-consistent polarized valence double zeta basis augmented with diffuse functions at the jun level | |
| CBS | [30] | complete basis set (limit obtained by extrapolation) | |
| Kinetics methods |  |  | |
| CVT | [31] | Canonical variational transition state theory | |
| SCT | [32] | Small-curvature tunneling | |

*^a^*We use the standard notation by which A/B denotes geometries and energies calculated by method A and basis set B, and A/B//C/E denotes single-point energies by A/B at geometries optimized by C/E. For composite methods, A/B is replaced by the name of the composite method.

Table S2. The scale factors for vibrational frequencies

| Method | Method abbreviation | Factor^[33]^ |
| --- | --- | --- |
| CCSD(T)-F12a/cc-pVTZ-F12 | T | 0.984 |
| CCSD(T)-F12a/cc-pVDZ-F12 | D | 0.983 |
| DF-CCSD(T)-F12b/jun-cc-pVDZ | junD | 0.981 |
| DF-CCSD(T)-F12b/$\mathrm{jun}^{'}$-cc-pVDZ | $\mathrm{jun}^{'}$D | 0.981 |
| M11-L/MG3S |  | 0.985 |
| M08-HX/MG3S |  | 0.973 |

**CH_2_OO reaction**

Table S3. Mean unsigned deviations of complexation energies, barrier heights, and reaction energies for the CH_2_OO + FCHO reaction (in kcal/mol)

| Methods | $\Delta E$ | | $V^{\ddagger}$ | | $\Delta E$ | |  |
| --- | --- | --- | --- | --- | --- | --- | --- |
|  | C1 | C2 | TS1 | TS2 | P1 | P2 | MUD-G |
| W2X//T | -8.19 | -7.68 | -6.32 | -4.68 | -48.08 | -49.72 | 0.00 |
| W2X//D | -8.22 | -7.72 | -6.28 | -4.63 | -48.12 | -49.78 | 0.04 |
| W2X//$\mathrm{jun}^{'}D$ | -8.15 | -7.65 | -6.29 | -4.64 | -48.15 | -49.82 | 0.05 |
| W2X//junD | -8.14 | -7.63 | -6.29 | -4.63 | -48.16 | -49.82 | 0.06 |

*^a^*$\Delta E$ is a reaction energy, $V^{\ddagger}$ is a barrier height. We use MUD-G for mean unsigned deviations due to geometry changes, so as not to confuse them with mean unsigned deviations (MUDs) of energies due to the method chosen for single-point energies.

*^b^*CX, TSX, and PX denote precursor complexes, transition states, and products of the CH_2_OO + FCHO reaction.

Table S4. Mean unsigned deviations of relative zero-point vibrational energies for the CH_2_OO + FCHO reaction (in kcal/mol)

| Methods | ${\Delta E}_{ZPE}$ | | ${\Delta E}_{ZPE}^{\ddagger}$ | | ${\Delta E}_{ZPE}$ | |  |
| --- | --- | --- | --- | --- | --- | --- | --- |
|  | C1 | C2 | TS1 | TS2 | P1 | P2 | MUD-GF |
| T | 1.73 | 1.72 | 2.50 | 2.55 | 5.69 | 5.76 | 0.00 |
| D | 1.66 | 1.48 | 2.49 | 2.53 | 5.67 | 5.74 | 0.06 |
| junD | 1.57 | 1.54 | 2.50 | 2.56 | 5.64 | 5.74 | 0.07 |
| $\mathrm{jun}^{'}$D | 1.58 | 1.45 | 2.51 | 2.57 | 5.67 | 5.77 | 0.08 |

*^a^*${\Delta E}_{ZPE}^{\ddagger}$ and ${\Delta E}_{ZPE}$ are changes in zero-point vibrational energy in going from reactants to the transition state and the product, respectively. We use MUD-GF for mean unsigned deviations due to geometry and frequency changes, so as not to confuse them with mean unsigned deviations (MUDs) of energies due to the method chosen for single-point energies.

*^b^*The CX, TSX and PX denote precursor complexes, transition states, and products of the CH_2_OO + FCHO reaction.

Table S5. The enthalpies of activation and reaction enthalpies for the CH_2_OO + FCHO reaction at 0 K (in kcal/mol)

| Methods | $\Delta H_{0}$ | | ${\Delta H}_{0}^{\ddagger}$ | | $\Delta H_{0}$ | |  |
| --- | --- | --- | --- | --- | --- | --- | --- |
|  | C1 | C2 | TS1 | TS2 | P1 | P2 | MUD-GF |
| W2X//T | -6.46 | -5.97 | -3.83 | -2.12 | -42.39 | -43.95 | 0.00 |
| W2X//D | -6.56 | -6.23 | -3.80 | -2.09 | -42.45 | -44.03 | 0.09 |
| W2X//$\mathrm{jun}^{'}$D | -6.57 | -6.21 | -3.79 | -2.07 | -42.48 | -44.05 | 0.10 |
| W2X//junD | -6.57 | -6.09 | -3.79 | -2.07 | -42.52 | -44.08 | 0.10 |

*^a^*${\Delta H}_{0}^{\ddagger}$ is the enthalpy of activations at 0 K and is given by $V^{\ddagger}+ {\Delta E}_{ZPE}^{\ddagger}$, and ${\Delta H}_{0}$ is the enthalpy of reaction at 0 K and is given by $\Delta E+{\Delta E}_{ZPE}$. We use MUD-GF for mean unsigned deviations due to geometry and frequency changes, so as not to confuse them with mean unsigned deviations (MUDs) of energies due to the method chosen for single-point energies.

*^b^*The CX, TSX and PX denote precursor complexes, transition states and products of the CH_2_OO + FCHO reaction.

**CH_2_OO, *anti*-CH_3_CHOO, and *syn*-CH_3_CHOO reactions**

Table S6. The enthalpies of activation (in kcal/mol) at 0 K for the reactions of FCHO with CH_2_OO, *anti*-CH_3_CHOO, and *syn*-CH_3_CHOO

| Method | ${\Delta H}_{0}^{\ddagger}$ | | | | | |  |
| --- | --- | --- | --- | --- | --- | --- | --- |
|  | TS1 | TS2 | a-TS1 | a-TS2 | s-TS1 | s-TS2 | MUD |
| MW3X-L//junD | -3.04 | -1.26 | -6.19 | -4.67 | -0.04 | 1.77 | 0.00 |
| W3X-L//junD | -3.23 | -1.46 | -6.42 | -4.90 | -0.32 | 1.5 | 0.23 |
| MW2-F12//junD | -3.59 | -1.87 | -6.79 | -5.32 | -0.59 | 1.19 | 0.59 |
| M11-L/MG3S | -3.89 | -2.02 | -6.83 | -5.31 | -0.81 | 0.75 | 0.78 |
| W2X//$\mathrm{jun}^{'}$D | -3.79 | -2.07 | -7.00 | -5.53 | -0.85 | 0.95 | 0.81 |
| W2X/junD | -3.79 | -2.07 | -7.01 | -5.54 | -0.87 | 0.92 | 0.82 |

*^a^*${\Delta H}_{0}^{\ddagger}$ is the enthalpy of activation at 0 K and is given by $V^{\ddagger}+ {\Delta E}_{ZPE}^{\ddagger}$.

*^b^*TS1, TS2, a-TS1, a-TS2, s-TS1, and s-TS2 denote transition states of the reactions of FCHO with CH_2_OO, *anti*-CH_3_CHOO, and *syn*-CH_3_CHOO, in which the prefixes “a” and “s” denote the “*anti*” and “*syn*” isomer, respectively.

Table S7. HPL rate constants (cm^3^ molecule^-1^ s^-1^) and transmission coefficients (unitless) for the FCHO + CH_2_OO reaction.

| *T*/K | $k_{\mathrm{TST}}^{\mathrm{HL}}$*^a^* | $\Gamma_{\mathrm{CVT}}^{\mathrm{LL}}$*^b^* | $\kappa_{\mathrm{SCT}}^{\mathrm{LL}}$*^c^* | $k_{CVT/SCT}^{\mathrm{DL}}$*^d^* | $F_{\mathrm{fwd}}^{MS-T}$*^e^* | ${k_{1}}_{MS-CVT/SCT}^{\mathrm{DL}}$*^f^* |
| --- | --- | --- | --- | --- | --- | --- |
| 190 | 4.79E-12 | 9.21E-01 | 1.32E+00 | 5.80E-12 | 0.80 | 4.67E-12 |
| 200 | 3.10E-12 | 9.20E-01 | 1.28E+00 | 3.65E-12 | 0.81 | 2.95E-12 |
| 210 | 2.10E-12 | 9.19E-01 | 1.25E+00 | 2.41E-12 | 0.81 | 1.96E-12 |
| 220 | 1.48E-12 | 9.18E-01 | 1.22E+00 | 1.66E-12 | 0.81 | 1.35E-12 |
| 230 | 1.08E-12 | 9.17E-01 | 1.20E+00 | 1.18E-12 | 0.82 | 9.69E-13 |
| 240 | 8.05E-13 | 9.15E-01 | 1.18E+00 | 8.72E-13 | 0.82 | 7.18E-13 |
| 250 | 6.18E-13 | 9.14E-01 | 1.17E+00 | 6.60E-13 | 0.83 | 5.46E-13 |
| 260 | 4.86E-13 | 9.12E-01 | 1.15E+00 | 5.12E-13 | 0.83 | 4.25E-13 |
| 270 | 3.90E-13 | 9.11E-01 | 1.14E+00 | 4.05E-13 | 0.84 | 3.39E-13 |
| 280 | 3.18E-13 | 9.09E-01 | 1.13E+00 | 3.27E-13 | 0.84 | 2.75E-13 |
| 290 | 2.64E-13 | 9.07E-01 | 1.12E+00 | 2.68E-13 | 0.85 | 2.27E-13 |
| 298 | 2.30E-13 | 9.05E-01 | 1.11E+00 | 2.32E-13 | 0.85 | 1.97E-13 |
| 300 | 2.22E-13 | 9.05E-01 | 1.11E+00 | 2.24E-13 | 0.85 | 1.90E-13 |
| 310 | 1.90E-13 | 9.03E-01 | 1.11E+00 | 1.89E-13 | 0.86 | 1.62E-13 |
| 320 | 1.64E-13 | 9.01E-01 | 1.10E+00 | 1.62E-13 | 0.86 | 1.39E-13 |
| 330 | 1.43E-13 | 8.99E-01 | 1.09E+00 | 1.40E-13 | 0.87 | 1.21E-13 |
| 340 | 1.26E-13 | 8.97E-01 | 1.09E+00 | 1.23E-13 | 0.87 | 1.07E-13 |
| 350 | 1.12E-13 | 8.95E-01 | 1.08E+00 | 1.08E-13 | 0.88 | 9.49E-14 |

*^a^*$k_{\mathrm{TST}}^{\mathrm{HL}}$ is the higher-level calculation by conventional transition state theory without a transmission coefficient.

*^b^*$\Gamma_{\mathrm{CVT}}^{\mathrm{LL}}$ is the lower-level recrossing transmission coefficient, which equals ${k_{\mathrm{CVT}}^{\mathrm{LL}}}/{k_{\mathrm{TST}}^{\mathrm{LL}}}$.

*^c^*$\kappa_{\mathrm{SCT}}^{\mathrm{LL}}$ is the lower-level tunneling transmission coefficient calculated by the small-curvature
tunneling approximation.

*^d^* The nonfinal high-pressure-limit (HPL) rate constant $k_{CVT/SCT}^{\mathrm{DL}}$ is calculated by the dual-level CVT/SCT method, by which it $\mathrm{equals} k_{\mathrm{TST}}^{\mathrm{HL}} \kappa_{\mathrm{SCT}}^{\mathrm{LL}}$ $\Gamma_{\mathrm{CVT}}^{\mathrm{LL}}$, where HL is MW3X-L//T, and LL is M11-L/MG3S for TS1 of the CH_2_OO + FCHO reaction.

*^e^*$F_{\mathrm{fwd}}^{MS-T}$ is the multistructural and torsional anharmonicity factor of the CH_2_OO + FCHO forward reaction.

*^d^*${k_{1}}_{MS-CVT/SCT}^{\mathrm{DL}}$ is the rate constant of CH_2_OO + FCHO reaction.

Table S8. HPL rate constants (cm^3^ molecule^-1^ s^-1^) and transmission coefficients (unitless) for the *anti*-CH_3_CHOO + FCHO reaction

| *T*/K | $k_{\mathrm{TST}}^{\mathrm{HL}}$*^a^* | $\Gamma_{\mathrm{CVT}}^{\mathrm{LL}}$*^b^* | $\kappa_{\mathrm{SCT}}^{\mathrm{LL}}$*^c^* | $k_{CVT/SCT}^{\mathrm{DL}}$*^d^* | *k*_VRC-TST_*^e^* | $F_{\mathrm{fwd}}^{MS-T}$*^f^* | ${k_{2}}_{\mathrm{CUS}}^{\mathrm{DL}}$*^g^* |
| --- | --- | --- | --- | --- | --- | --- | --- |
| 190 | 1.15E-08 | 9.09E-01 | 1.19E+00 | 1.24E-08 | 8.69E-10 | 0.63 | 7.82E-10 |
| 200 | 4.97E-09 | 9.08E-01 | 1.17E+00 | 5.26E-09 | 8.48E-10 | 0.64 | 6.76E-10 |
| 210 | 2.34E-09 | 9.06E-01 | 1.15E+00 | 2.44E-09 | 8.28E-10 | 0.64 | 5.41E-10 |
| 220 | 1.18E-09 | 9.05E-01 | 1.14E+00 | 1.22E-09 | 8.08E-10 | 0.64 | 3.97E-10 |
| 230 | 6.37E-10 | 9.04E-01 | 1.12E+00 | 6.47E-10 | 7.72E-10 | 0.65 | 2.71E-10 |
| 240 | 3.62E-10 | 9.02E-01 | 1.11E+00 | 3.63E-10 | 7.41E-10 | 0.65 | 1.79E-10 |
| 250 | 2.16E-10 | 9.00E-01 | 1.10E+00 | 2.14E-10 | 7.15E-10 | 0.65 | 1.17E-10 |
| 260 | 1.34E-10 | 8.98E-01 | 1.09E+00 | 1.32E-10 | 6.93E-10 | 0.66 | 7.72E-11 |
| 270 | 8.68E-11 | 8.96E-01 | 1.09E+00 | 8.46E-11 | 6.73E-10 | 0.66 | 5.16E-11 |
| 280 | 5.80E-11 | 8.94E-01 | 1.08E+00 | 5.61E-11 | 6.56E-10 | 0.67 | 3.53E-11 |
| 290 | 3.99E-11 | 8.92E-01 | 1.08E+00 | 3.83E-11 | 6.42E-10 | 0.67 | 2.46E-11 |
| 298 | 3.02E-11 | 8.90E-01 | 1.07E+00 | 2.88E-11 | 6.31E-10 | 0.67 | 1.88E-11 |
| 300 | 2.83E-11 | 8.90E-01 | 1.07E+00 | 2.69E-11 | 6.29E-10 | 0.67 | 1.76E-11 |
| 310 | 2.05E-11 | 8.88E-01 | 1.07E+00 | 1.94E-11 | 6.17E-10 | 0.68 | 1.28E-11 |
| 320 | 1.52E-11 | 8.85E-01 | 1.06E+00 | 1.43E-11 | 6.07E-10 | 0.68 | 9.57E-12 |
| 330 | 1.15E-11 | 8.83E-01 | 1.06E+00 | 1.07E-11 | 5.98E-10 | 0.69 | 7.26E-12 |
| 340 | 8.84E-12 | 8.81E-01 | 1.05E+00 | 8.21E-12 | 5.91E-10 | 0.69 | 5.60E-12 |
| 350 | 6.93E-12 | 8.78E-01 | 1.05E+00 | 6.39E-12 | 5.83E-10 | 0.69 | 4.40E-12 |

*^a^*$k_{\mathrm{TST}}^{\mathrm{HL}}$ is the higher-level calculation by conventional transition state theory without a transmission coefficient.

*^b^*$\Gamma_{\mathrm{CVT}}^{\mathrm{LL}}$ is the lower-level recrossing transmission coefficient, which equals ${k_{\mathrm{CVT}}^{\mathrm{LL}}}/{k_{\mathrm{TST}}^{\mathrm{LL}}}$.

*^c^*$\kappa_{\mathrm{SCT}}^{\mathrm{LL}}$ is the lower-level tunneling transmission coefficient calculated by the small-curvature
tunneling approximation.

*^d^* The nonfinal HPL rate constant $k_{CVT/SCT}^{\mathrm{DL}}$ is calculated by the dual-level CVT/SCT method. Therefore $k_{a-TS1}^{\mathrm{DL}} \mathrm{equals} k_{\mathrm{TST}}^{L1} \kappa_{\mathrm{SCT}}^{L2}$ $\Gamma_{\mathrm{CVT}}^{L2}$, where L1 is MW3X-L//junD, and L2 is M11-L/MG3S for transition state 1 of the *anti*-CH_3_CHOO + FCHO reaction.

*^e^ k*_VRC-TST_ is calculated by M11-L/MG3S for the loose transition state of the *anti*-CH_3_CHOO + FCHO reaction.

*^f^*$F_{\mathrm{fwd}}^{MS-T}$ is the multistructural and torsional anharmonicity factor of *anti*-CH_3_CHOO + FCHO forward reaction.

*^g^*${k_{2}}_{\mathrm{CUS}}^{\mathrm{DL}}$ is the rate constant of *anti*-CH_3_CHOO + FCHO reaction, given by
 ${{k_{2}}_{\mathrm{CUS}}^{\mathrm{DL}}=(F}_{\mathrm{fwd}}^{MS-T}k_{CVT/SCT}^{\mathrm{DL}}$*k*_VRC-TST_)/(${F_{\mathrm{fwd}}^{MS-T} k}_{CVT/SCT}^{\mathrm{DL}}$+ *k*_VRC-TST_).

Table S9. HPL rate constants (cm^3^ molecule^-1^ s^-1^) and transmission coefficients (unitless) for the *syn*-CH_3_CHOO + FCHO reaction

| *T*/K | $k_{\mathrm{TST}}^{\mathrm{HL}}$*^a^* | $\Gamma_{\mathrm{CVT}}^{\mathrm{LL}}$*^b^* | $\kappa_{\mathrm{SCT}}^{\mathrm{LL}}$*^c^* | $k_{CVT/SCT}^{\mathrm{DL}}$*^d^* | $F_{\mathrm{fwd}}^{MS-T}$*^e^* | ${k_{3}}_{MS-CVT/SCT}^{\mathrm{DL}}$*^f^* |
| --- | --- | --- | --- | --- | --- | --- |
| 190 | 5.37E-16 | 8.97E-01 | 1.29E+00 | 6.19E-16 | 0.91 | 5.61E-16 |
| 200 | 5.26E-16 | 8.96E-01 | 1.25E+00 | 5.91E-16 | 0.89 | 5.25E-16 |
| 210 | 5.18E-16 | 8.94E-01 | 1.23E+00 | 5.68E-16 | 0.87 | 4.96E-16 |
| 220 | 5.12E-16 | 8.93E-01 | 1.20E+00 | 5.50E-16 | 0.86 | 4.72E-16 |
| 230 | 5.09E-16 | 8.91E-01 | 1.18E+00 | 5.36E-16 | 0.84 | 4.53E-16 |
| 240 | 5.07E-16 | 8.89E-01 | 1.17E+00 | 5.26E-16 | 0.83 | 4.37E-16 |
| 250 | 5.07E-16 | 8.86E-01 | 1.15E+00 | 5.18E-16 | 0.82 | 4.23E-16 |
| 260 | 5.08E-16 | 8.84E-01 | 1.14E+00 | 5.12E-16 | 0.81 | 4.12E-16 |
| 270 | 5.10E-16 | 8.82E-01 | 1.13E+00 | 5.08E-16 | 0.79 | 4.03E-16 |
| 280 | 5.14E-16 | 8.79E-01 | 1.12E+00 | 5.05E-16 | 0.78 | 3.95E-16 |
| 290 | 5.18E-16 | 8.76E-01 | 1.11E+00 | 5.04E-16 | 0.77 | 3.89E-16 |
| 298 | 5.22E-16 | 8.74E-01 | 1.10E+00 | 5.04E-16 | 0.76 | 3.85E-16 |
| 300 | 5.23E-16 | 8.74E-01 | 1.10E+00 | 5.04E-16 | 0.76 | 3.84E-16 |
| 310 | 5.30E-16 | 8.71E-01 | 1.10E+00 | 5.06E-16 | 0.75 | 3.79E-16 |
| 320 | 5.37E-16 | 8.68E-01 | 1.09E+00 | 5.08E-16 | 0.74 | 3.76E-16 |
| 330 | 5.44E-16 | 8.65E-01 | 1.08E+00 | 5.11E-16 | 0.73 | 3.73E-16 |
| 340 | 5.53E-16 | 8.62E-01 | 1.08E+00 | 5.14E-16 | 0.72 | 3.71E-16 |
| 350 | 5.62E-16 | 8.59E-01 | 1.07E+00 | 5.19E-16 | 0.71 | 3.70E-16 |

*^a^*$k_{\mathrm{TST}}^{\mathrm{HL}}$ is the higher-level calculation by conventional transition state theory without a transmission coefficient.

*^b^*$\Gamma_{\mathrm{CVT}}^{\mathrm{LL}}$ is the lower-level recrossing transmission coefficient, which equals ${k_{\mathrm{CVT}}^{L2}}/{k_{\mathrm{TST}}^{L2}}$.

*^c^*$\kappa_{\mathrm{SCT}}^{\mathrm{LL}}$ is the lower-level tunneling transmission coefficient calculated by the small-curvature
tunneling approximation.

*^d^* The nonfinal high-pressure-limit (HPL) rate constant $k_{CVT/SCT}^{\mathrm{DL}}$ is calculated by the dual-level CVT/SCT method, by which it $\mathrm{equals} k_{\mathrm{TST}}^{\mathrm{HL}} \kappa_{\mathrm{SCT}}^{\mathrm{LL}}$ $\Gamma_{\mathrm{CVT}}^{\mathrm{LL}}$, where HL is MW3X-L//junD, and LL is M11-L/MG3S for transition state 1 of the *syn*-CH_3_CHOO + FCHO reaction.

*^e^*$F_{\mathrm{fwd}}^{MS-T}$ is the multistructural and torsional anharmonicity factor of the *syn*-CH_3_CHOO + FCHO forward reaction.

*^f^*${k_{3}}_{MS-CVT/SCT}^{\mathrm{DL}}$ is the rate constant of *syn*-CH_3_CHOO + FCHO, equal to $k_{CVT/SCT}^{\mathrm{DL}}$ $F_{\mathrm{fwd}}^{MS-T}$.

Table S10. Fitting parameters of high-pressure-limit rate constants for forward reaction

|  | ln *A*  *(A* in s^–1^) | *n*  (unitless) | *E*  (kcal/mol) | *T*_0_  (K) |
| --- | --- | --- | --- | --- |
| CH_2_OO + FCHO | -27.09 | -4.75 | 3.54 | -88.42 |
| *anti*-CH_3_CHOO + FCHO | -103.43 | 46.99 | -63.24 | -48.04 |
| *syn*-CH_3_CHOO + FCHO | -37.53 | 1.58 | -1.29 | -10.24 |

**CH_2_OO, OH, and HO_2_ reactions**

Table S11. The ratio between CH_2_OO + FCHO and FCHO + OH reaction rates for various concentrations in molecules/cm^3^

| ***T*** | ***k*_1_** | ***k_OH_*** | $\boldsymbol{v}_{\boldsymbol{1}}^{\boldsymbol{a}}$ | | | | | |
| --- | --- | --- | --- | --- | --- | --- | --- | --- |
|  |  |  | **[CH_2_OO]=10^4^** | | | **[CH_2_OO]=10^5^** | | |
|  |  |  | **[OH]**  **=10^4^** | **[OH]**  **=10^5^** | **[OH]**  **=10^6^** | **[OH]**  **=10^4^** | **[OH]**  **=10^5^** | **[OH]**  **=10^6^** |
| 190 | 4.67E-12 | 1.30E-16 | 3.60E+04 | 3.60E+03 | 3.60E+02 | 3.60E+05 | 3.60E+04 | 3.60E+03 |
| 200 | 2.95E-12 | 1.80E-16 | 1.64E+04 | 1.64E+03 | 1.64E+02 | 1.64E+05 | 1.64E+04 | 1.64E+03 |
| 210 | 1.96E-12 | 2.44E-16 | 8.04E+03 | 8.04E+02 | 8.04E+01 | 8.04E+04 | 8.04E+03 | 8.04E+02 |
| 220 | 1.35E-12 | 3.24E-16 | 4.17E+03 | 4.17E+02 | 4.17E+01 | 4.17E+04 | 4.17E+03 | 4.17E+02 |
| 230 | 9.69E-13 | 4.24E-16 | 2.29E+03 | 2.29E+02 | 2.29E+01 | 2.29E+04 | 2.29E+03 | 2.29E+02 |
| 240 | 7.18E-13 | 5.45E-16 | 1.32E+03 | 1.32E+02 | 1.32E+01 | 1.32E+04 | 1.32E+03 | 1.32E+02 |
| 250 | 5.46E-13 | 6.91E-16 | 7.90E+02 | 7.90E+01 | 7.90E+00 | 7.90E+03 | 7.90E+02 | 7.90E+01 |
| 260 | 4.25E-13 | 8.64E-16 | 4.92E+02 | 4.92E+01 | 4.92E+00 | 4.92E+03 | 4.92E+02 | 4.92E+01 |
| 270 | 3.39E-13 | 1.07E-15 | 3.17E+02 | 3.17E+01 | 3.17E+00 | 3.17E+03 | 3.17E+02 | 3.17E+01 |
| 280 | 2.75E-13 | 1.31E-15 | 2.10E+02 | 2.10E+01 | 2.10E+00 | 2.10E+03 | 2.10E+02 | 2.10E+01 |
| 290 | 2.27E-13 | 1.58E-15 | 1.44E+02 | 1.44E+01 | 1.44E+00 | 1.44E+03 | 1.44E+02 | 1.44E+01 |
| 298 | 1.97E-13 | 1.83E-15 | 1.08E+02 | 1.08E+01 | 1.08E+00 | 1.08E+03 | 1.08E+02 | 1.08E+01 |
| 300 | 1.90E-13 | 1.89E-15 | 1.01E+02 | 1.01E+01 | 1.01E+00 | 1.01E+03 | 1.01E+02 | 1.01E+01 |
| 310 | 1.62E-13 | 2.25E-15 | 7.19E+01 | 7.19E+00 | 7.19E-01 | 7.19E+02 | 7.19E+01 | 7.19E+00 |
| 320 | 1.39E-13 | 2.66E-15 | 5.25E+01 | 5.25E+00 | 5.25E-01 | 5.25E+02 | 5.25E+01 | 5.25E+00 |
| 330 | 1.21E-13 | 3.11E-15 | 3.90E+01 | 3.90E+00 | 3.90E-01 | 3.90E+02 | 3.90E+01 | 3.90E+00 |
| 340 | 1.07E-13 | 3.62E-15 | 2.95E+01 | 2.95E+00 | 2.95E-01 | 2.95E+02 | 2.95E+01 | 2.95E+00 |
| 350 | 9.49E-14 | 4.19E-15 | 2.26E+01 | 2.26E+00 | 2.26E-01 | 2.26E+02 | 2.26E+01 | 2.26E+00 |

*^a^*$v_{1}=\frac{v}{v_{\mathrm{OH}}}=\frac{k_{1}\left[ \mathrm{FCHO} \right][{CH}_{2}OO]}{k_{\mathrm{OH}}\left[ \mathrm{FCHO} \right][OH]}=\frac{k_{1}[{CH}_{2}OO]}{k_{\mathrm{OH}}[OH]}$ , where *k*_1_ is the rate constant of CH_2_OO + FCHO, and *k*_OH_ is the rate constant of FCHO + OH. Both *k*_1_ and *k*_OH_ are from this work.

Table S12. Atmospheric lifetimes of FCHO for reaction with CH_2_OO, OH, and HO_2_ as functions of temperature

| T (K) | τ_1_*^a,b^*(s) | τ_2_*^c^*(s) | τ_3_*^d^*(s) |
| --- | --- | --- | --- |
| 220 | 7.40E+07 | 3.09E+09 | 2.35E+09 |
| 230 | 1.03E+08 | 2.36E+09 | 1.78E+09 |
| 240 | 1.39E+08 | 1.84E+09 | 1.37E+09 |
| 250 | 1.83E+08 | 1.45E+09 | 1.07E+09 |
| 260 | 2.35E+08 | 1.16E+09 | 8.51E+08 |
| 270 | 2.95E+08 | 9.37E+08 | 6.86E+08 |
| 280 | 3.64E+08 | 7.66E+08 | 5.60E+08 |
| 290 | 4.41E+08 | 6.33E+08 | 4.62E+08 |
| **298** | **5.08E+08** | **5.47E+08** | **3.99E+08** |
| 300 | 5.25E+08 | 5.28E+08 | 3.85E+08 |
| 310 | 6.18E+08 | 4.44E+08 | 3.24E+08 |
| 320 | 7.17E+08 | 3.76E+08 | 2.75E+08 |

*^a^*$\tau=\frac{1}{k_{b}[X]}$ , where *k*_b_ is the rate constant for reaction with X, and [X] stands for the concentration of X, where X is CH_2_OO, OH, or HO_2_.

*^b^*τ_1_ is the reaction-specific atmospheric lifetime for the FCHO + CH_2_OO reaction, where the concentration of CH_2_OO is 1 × 10^4^ molecules/cm^3^.

*^c^*τ_2_ is the reaction-specific atmospheric lifetime for the FCHO + OH reaction, where the concentration of OH is 1 × 10^6^ molecules/cm^3^.

*^d^*τ_3_ is the reaction-specific atmospheric lifetime for the FCHO + HO_2_ reaction, where the concentration of HO_2_ is taken to be 1.1 × 10^9^ molecules/cm^3^. The concentration of HO_2_ varies greatly between day and night [Brasseur, G. P.; Solomon, S., *Aeronomy of the middle atmosphere: Chemistry and physics of the stratosphere and mesosphere*. Springer Science & Business Media: 2006; pp. 617-621], but this value is close to the maximum, which reached during the daytime [Lew, M. M.; Rickly, P. S.; Bottorff, B. P.; Reidy, E.; Sklaveniti, S.; Léonardis, T.; Locoge, N.; Dusanter, S.; Kundu, S.; Wood, E.; Stevens, P. S., OH and HO_2_ radical chemistry in a midlatitude forest: measurements and model comparisons. *Atmos. Chem. Phys.* **2020***, 20*, 9209-9230.].

**Geometries and absolute energies**

Table S13. Cartesian coordinates (Å) of optimized structures

| Species | Methods | Cartesian coordinates |
| --- | --- | --- |
| FCHO | D | O -0.2449314424 1.1272032214 0.0000000000  C 0.3803112035 0.1260065247 0.0000000000  F -0.1940302926 -1.0875129032 0.0000000000  H 1.4664873714 0.0151688171 0.0000000000 |
|  | junD | O -0.2499534360 1.1318226741 0.0000000000  C 0.3803327150 0.1283125744 0.0000000000  F -0.1983647049 -1.0932995998 0.0000000000  H 1.4758222658 0.0140300114 0.0000000000 |
|  | M11-L/MG3S | O 1.12470500 0.10445300 0.00000000  C 0.00000000 0.38317200 0.00000000  H -0.44619300 1.39893700 0.00000000  F -0.95016100 -0.50373200 0.00000000 |
| CH_2_OO | D | C 1.0738898213 -0.2040618152 0.0000000000  O -0.0046664679 0.4706154646 0.0000000000  O -1.1647544521 -0.2048945578 0.0000000000  H 1.0101594984 -1.2843811341 0.0000000000  H 1.9777416003 0.3858980425 0.0000000000 |
|  | junD | C 1.0745904807 -0.2044064377 0.0000000000  O -0.0081258289 0.4706452826 0.0000000000  O -1.1695901229 -0.2018571835 0.0000000000  H 1.0109079706 -1.2948993528 0.0000000000  H 1.9845875005 0.3936936913 0.0000000000 |
|  | M11-L/MG3S | C 1.03953700 -0.21812800 0.00000000  H 0.97611200 -1.31070200 0.00000000  H 1.96189500 0.36313000 0.00000000  O 0.00000000 0.45058600 0.00000000  O -1.14690400 -0.16854300 0.00000000 |
| C1 | D | C -1.5995823444 0.6845030673 0.5686926670  O -1.6545667671 -0.2342636325 -0.2963944574  O -0.7667920618 -1.2557872763 -0.1534724487  H -0.9095103099 0.5913926577 1.3961081252  H -2.2884052235 1.5014942132 0.4086326928  O 0.8037725762 1.1759849005 -0.4388828832  C 1.2863245992 0.0884662970 -0.4446054742  F 1.8958731971 -0.4130153036 0.6381012568  H 1.3776283341 -0.5965269233 -1.2841224783 |
|  | junD | C -1.6248334179 0.6902554940 0.5757118781  O -1.6580847551 -0.2295900756 -0.2944284116  O -0.7666250135 -1.2471500641 -0.1406587736  H -0.9400437912 0.5992695630 1.4213702167  H -2.3260876703 1.5073821286 0.4041214691  O 0.8230713770 1.1732762830 -0.4362339945  C 1.3099632284 0.0830486731 -0.4566269874  F 1.9274138425 -0.4298266316 0.6279767393  H 1.3999682000 -0.6044173705 1.3071751362 |
|  | M11-L/MG3S | C -1.56667800 0.66847300 0.55568600  O -1.60834200 -0.22842300 -0.28741200  O -0.73450400 -1.21285600 -0.15373600  H -0.87506000 0.59321700 1.39956000  H -2.26829600 1.48926900 0.39249200  O 0.75877700 1.14927900 -0.42450100  C 1.24539100 0.08461800 -0.42716200  F 1.81877200 -0.40796200 0.62785800  H 1.37468200 -0.59336700 -1.28872800 |
| C2 | D | C -1.7339281780 0.7265537684 -0.2068419663  O -1.4026009796 -0.4770249566 -0.3683532104  O -0.8348273859 -1.1011801178 0.6905935777  H -1.5943523045 1.1879024980 0.7629712781  H -2.1580865657 1.2025212675 -1.0813023851  O 0.8609265225 1.2689139940 0.3270477418  C 1.3137244835 0.1745656107 0.3936778301  H 1.5470982867 -0.3974604206 1.2914241864  F 1.6875461210 -0.4912976436 -0.6976360523 |
|  | junD | C -1.7590028014 0.7343919767 -0.2054220596  O -1.4103239817 -0.4715082243 -0.3775154421  O -0.8367391907 -1.1028336411 0.6829081832  H -1.6280997819 1.1905905317 0.7775309669  H -2.1859146825 1.2152413614 -1.0860099232  O 0.8688682091 1.2655580307 0.3093204232  C 1.3378417327 0.1717314113 0.3984387694  H 1.5736146266 -0.3953303209 1.3077826857  F 1.7252558696 -0.5143471253 -0.6954526037 |
|  | M11-L/MG3S | C -1.72862000 0.72269300 -0.17558900  O -1.39051700 -0.44123700 -0.39326700  O -0.79177500 -1.09017000 0.59165700  H -1.57045700 1.16008200 0.81419800  H -2.18988400 1.23404400 -1.02323400  O 0.82194500 1.22260400 0.19739100  C 1.29320500 0.16662300 0.37202600  H 1.48985400 -0.33383900 1.33642700  F 1.75174900 -0.54730600 -0.60802800 |
| TS1 | D | C -1.3851398791 0.6287517021 0.5658011447  O -1.5114211181 -0.3491184139 -0.2287188276  O -0.4583646119 -1.2368438951 -0.1199863912  H -0.7313859596 0.5442067320 1.4211093333  H -2.0965212087 1.4302008113 0.4173843231  O 0.4873747267 1.1591389641 -0.4556973036  C 1.0451439010 0.0792712412 -0.4588876062  F 1.7784138090 -0.2980065533 0.6084703211  H 1.3246783409 -0.4892165884 -1.3407569938 |
|  | junD | C -1.3881817273 0.6329600587 0.5665466724  O -1.5080755328 -0.3503019975 -0.2288317746  O -0.4435219906 -1.2272024860 -0.1141540789  H -0.7410144390 0.5460403040 1.4397802879  H -2.1101707349 1.4372767858 0.4106499282  O 0.4844444698 1.1595294702 -0.4516033497  C 1.0403967492 0.0713512141 -0.4645030360  F 1.7847076234 -0.3097611747 0.6085695943  H 1.3341935824 -0.4915081746 -1.3577362439 |
|  | M11-L/MG3S | C -1.36166600 0.60731900 0.56162500  O -1.48506300 -0.33643700 -0.23012800  O -0.45326400 -1.19524300 -0.14079300  H -0.68436700 0.52546600 1.41425900  H -2.07715600 1.42398000 0.42996000  O 0.46429600 1.13709500 -0.47799700  C 1.01570800 0.07768600 -0.44307600  F 1.69940400 -0.26641400 0.61762000  H 1.33488600 -0.50506800 -1.32275200 |
| TS2 | D | C -1.5955359747 0.5752348666 -0.1609440313  O -1.1966557092 -0.5994368607 -0.4104926439  O -0.4251922175 -1.1006514218 0.6281949616  H -1.6359315665 0.9232527069 0.8594003139  H -2.0540911033 1.0837201577 -0.9993054236  O 0.4485485720 1.2715943621 0.1597180630  C 1.0389557946 0.2289901330 0.3894221515  H 1.4513688741 -0.0696717809 1.3492037073  F 1.6764833303 -0.3964671631 -0.6200230983 |
|  | junD | C -1.6001650886 0.5770755341 -0.1652893570  O -1.1921811579 -0.5995895680 -0.4113717338  O -0.4135847165 -1.0875840300 0.6312972036  H -1.6530751023 0.9282714283 0.8642988036  H -2.0644769757 1.0824273472 -1.0154330679  O 0.4498177351 1.2748311401 0.1707473641  C 1.0384011540 0.2231328901 0.3963333344  H 1.4728199260 -0.0798683884 1.3566001136  F 1.6703942257 -0.4021313536 -0.6320086603 |
|  | M11-L/MG3S | C -1.58333600 0.55660700 -0.15993300  O -1.17094800 -0.58278500 -0.40714100  O -0.41275100 -1.05780500 0.60758700  H -1.60838600 0.92250800 0.86763700  H -2.05733000 1.06995700 -1.00280100  O 0.44576600 1.25197800 0.15689700  C 1.01765600 0.22191000 0.37911600  H 1.44153100 -0.07969600 1.35287300  F 1.63574800 -0.38610900 -0.59906100 |
| P1 | D | C 1.1567201087 -0.6451932152 0.3749562998  O 1.3877622205 0.6895504982 0.0159624591  O 0.0180962749 1.1766784789 -0.1080804785  H 0.8469710113 -0.7279712660 1.4200060258  H 2.0482556657 -1.2204883371 0.1432334697  O 0.0997993656 -1.0352352006 -0.4857952174  C -0.7475829688 0.0402879744 -0.4538132473  F -1.6963115812 -0.1029895977 0.5181371485  H -1.2601490966 0.1699746650 -1.4033004595 |
|  | junD | C 1.1547105726 -0.6460968817 0.3779609274  O 1.3914583145 0.6915288900 0.0251850376  O 0.0196086233 1.1836747392 -0.1275376635  H 0.8326034632 -0.7366939221 1.4280661915  H 2.0564849529 -1.2228500940 0.1472997315  O 0.1062189130 -1.0308898290 -0.4983808622  C -0.7450810263 0.0432286603 -0.4595462862  F -1.6830707170 -0.1066011531 0.5355203136  H -1.2793720960 0.1693135904 -1.4072613897 |
|  | M11-L/MG3S | C 1.14479100 -0.62823600 0.35647600  O 1.33792000 0.66200600 -0.03713600  O 0.00769600 1.12051000 -0.01073100  H 0.90691900 -0.69663800 1.43686500  H 2.03720500 -1.21368700 0.09988900  O 0.07170100 -1.03291700 -0.41419900  C -0.74186100 0.03672000 -0.42725400  F -1.72368000 -0.07928000 0.44588100  H -1.18713000 0.17613600 -1.42848500 |
| P2 | D | C 1.3639310338 -0.4879496545 -0.0884695162  O 1.0037729888 0.8107215243 -0.4817025962  O -0.0353060297 1.0722631707 0.5129323045  H 1.9969273370 -0.4729069588 0.8017868370  H 1.8399410756 -0.9674967666 -0.9406965014  O 0.1338894413 -1.1312604279 0.2168759564  C -0.7672931071 -0.1083897861 0.4437204978  H -1.3123522036 -0.2212853012 1.3786993148  F -1.6661455360 -0.0704887999 -0.5754312966 |
|  | junD | C 1.3660847434 -0.4881551515 -0.0887137273  O 1.0009420311 0.8109639243 -0.4816608846  O -0.0381831285 1.0736380024 0.5197515382  H 2.0141220215 -0.4738381409 0.8020312734  H 1.8354401987 -0.9696591103 -0.9549677110  O 0.1363577741 -1.1320588350 0.2307040531  C -0.7681715770 -0.1083175481 0.4473087922  H -1.3295525661 -0.2188844792 1.3828391854  F -1.6596744973 -0.0704816617 -0.5895775194 |
|  | M11-L/MG3S | C 1.32834200 -0.47510500 -0.08602100  O 0.97459900 0.78197100 -0.47872400  O -0.02357800 1.03950800 0.49931000  H 1.96966100 -0.46364200 0.81843000  H 1.83985100 -0.96064300 -0.93022400  O 0.12980700 -1.10645700 0.18791200  C -0.74827400 -0.10639600 0.42607300  H -1.28510500 -0.22092300 1.38439200  F -1.62793800 -0.06510600 -0.55343300 |
| anti-CH3CHOO | junD | C 0.3704518992 0.4184433374 -0.0000000761  H 0.0911760199 1.4784462126 -0.0000009663  O -0.5888447874 -0.4152422930 0.0000008414  O -1.8591575291 0.0807745342 0.0000007607  C 1.7611424524 -0.1042547661 -0.0000003224  H 2.2981983851 0.2619546385 0.8885423023  H 1.7550431272 -1.2011347933 0.0000006078  H 2.2981974328 0.2619531298 -0.8885441473 |
|  | M11-L/MG3S | C 0.36745900 0.41173700 0.00000100  H 0.10037500 1.47711000 0.00000000  O -0.56926600 -0.39831400 0.00000000  O -1.80367300 0.06492100 0.00000000  C 1.72941400 -0.10117500 0.00000000  H 2.28317200 0.26041200 0.87709600  H 1.73555600 -1.19416000 0.00000100  H 2.28317000 0.26040900 -0.87709900 |
| syn-CH3CHOO | junD | C 0.4645323302 0.7116650091 -0.0000576727  H 0.7819946006 1.7570510893 -0.0001240744  O -0.8067312833 0.5844127044 0.0000118267  O -1.2900137616 -0.6865312829 0.0000974961  C 1.3442666820 -0.4720776540 -0.0000440486  H 1.1015765248 -1.0938989883 -0.8776538241  H 2.3993210190 -0.1761770526 -0.0001109210  H 1.1016638882 -1.0938138251 0.8776502181 |
|  | M11-L/MG3S | C 0.45800100 0.69939300 -0.00005700  H 0.77767500 1.74508000 -0.00012300  O -0.77870600 0.56788600 0.00001100  O -1.22442000 -0.67688100 0.00009400  C 1.32705400 -0.45292600 -0.00004400  H 1.07733600 -1.08735500 -0.86396700  H 2.38224800 -0.17729600 -0.00011000  H 1.07742200 -1.08727100 0.86396500 |
| anti-C1 | junD | C 1.3048149335 0.0299019969 0.4405024116  O 0.9567633917 0.9686131892 -0.3314992745  O -0.2527527786 1.5744932770 -0.0331051970  H 0.6688891545 -0.1543184637 1.3128229911  O -0.7794525206 -1.1939815740 -0.5334461952  C -1.6297688265 -0.3554860184 -0.4486811864  F -2.3484465930 -0.2048201272 0.6841531833  H -2.0179198297 0.2983596481 -1.2385556195  C 2.5409849163 -0.7266019608 0.1279043775  H 3.2309924041 -0.6813831520 0.9837394138  H 2.2696773447 -1.7812540230 -0.0346071404  H 3.0234224035 -0.3242187921 -0.7707857644 |
|  | M11-L/MG3S | C 1.26987300 0.03735500 0.43054500  O 0.93187800 0.95622400 -0.32392800  O -0.24799300 1.53048500 -0.05001200  H 0.63347600 -0.15423400 1.30335400  O -0.70229500 -1.15719500 -0.51626700  C -1.54937500 -0.35030600 -0.42155300  F -2.22817700 -0.20512600 0.67622500  H -1.96984200 0.27896900 -1.22346500  C 2.47691900 -0.71002800 0.12365200  H 3.16979500 -0.69858100 0.97386100  H 2.21008400 -1.76143400 -0.04731900  H 2.97286100 -0.31682500 -0.76665100 |
| anti-C2 | junD | C -1.4402834312 0.0744433086 -0.3485109964  O -0.7811590142 0.9384159688 0.2973408571  O 0.2036969375 1.6055217200 -0.4117306078  H -1.2205969876 -0.0150148863 -1.4172609581  O 0.8531782856 -1.2052858494 -0.6913703059  C 1.6596730559 -0.3816320991 -0.3726783965  H 2.2656471112 0.2628475793 -1.0203389814  F 2.0164652085 -0.2235477090 0.9179400993  C -2.4543879289 -0.7311224980 0.3737462578  H -3.4385662097 -0.6013248962 -0.1012172124  H -2.1768454716 -1.7934923124 0.2933762604  H -2.5010665555 -0.4372883263 1.4291289839 |
|  | M11-L/MG3S | C -1.41165600 0.07763700 -0.33509700  O -0.77389900 0.90981100 0.31801700  O 0.20722500 1.54805200 -0.33602600  H -1.17410800 -0.00818700 -1.40260400  O 0.78387000 -1.18673700 -0.54712200  C 1.60354800 -0.37219000 -0.34883800  H 2.15158100 0.22858300 -1.09402900  F 2.08114600 -0.16764700 0.83941100  C -2.42295100 -0.71529100 0.34237700  H -3.39864600 -0.60026300 -0.14606600  H -2.15854200 -1.77789200 0.26100300  H -2.50181300 -0.44335600 1.39739900 |
| syn-C1 | junD | C 1.7133928837 0.0907267354 0.4958400972  O 1.2272500856 -1.0468862431 0.2056518591  O 0.4296762237 -1.0956105536 -0.9126517949  H 2.3138116861 0.0626321364 1.4095221633  O -1.1434812755 0.1661528115 1.2891110749  C -1.6578678299 -0.2515694674 0.2988013238  F -2.0150774206 0.5773313079 -0.7102503736  H -1.9640431057 -1.2795622089 0.0679856457  C 1.5142164252 1.2730477614 -0.3651647596  H 0.4450346437 1.5299221295 -0.3979663471  H 2.1045502565 2.1194463896 0.0036091057  H 1.7960604272 0.9981692011 -1.3950229944 |
|  | M11-L/MG3S | C 1.68933800 0.08051700 0.48096000  O 1.18711700 -1.01388000 0.18624000  O 0.41232700 -1.01128700 -0.90134900  H 2.29661000 0.03238700 1.39075300  O -1.12501000 0.22312600 1.23532000  C -1.62446800 -0.23760700 0.28700800  F -1.99740800 0.49791900 -0.72022400  H -1.91513800 -1.28906300 0.11459200  C 1.51406800 1.25451700 -0.34179800  H 0.44945800 1.51894500 -0.38471000  H 2.10412200 2.09693400 0.02079600  H 1.77250700 0.99129200 -1.37812300 |
| syn-C2 | junD | C -1.5726321506 -0.0699480615 -0.7026303299  O -0.7561803259 -1.0049689257 -0.4274915357  O -0.3865176042 -1.1219613884 0.8898203033  H -1.8013900890 -0.0224549746 -1.7709957149  O 1.0280772449 1.3653410207 -0.0956479680  C 1.6434761660 0.4536956857 0.3629187953  H 1.8239316694 0.2155295860 1.4189624369  F 2.2790262156 -0.4340479887 -0.4278893762  C -2.1494683942 0.7977758834 0.3419048993  H -1.3371907428 1.3642444909 0.8226552264  H -2.8948288009 1.4754910225 -0.0892332744  H -2.5876891883 0.1556946499 1.1233515381 |
|  | M11-L/MG3S | C -1.56166500 -0.06155900 -0.69928600  O -0.76259400 -0.98082900 -0.46524300  O -0.37935600 -1.09772500 0.80786800  H -1.82610800 0.00467400 -1.75966100  O 0.99675900 1.29757700 -0.20824700  C 1.57640500 0.44973000 0.34577200  H 1.68257900 0.30071800 1.43527100  F 2.26533400 -0.44242400 -0.29443500  C -2.09373000 0.77819700 0.34733700  H -1.26559400 1.31699000 0.82767100  H -2.84174500 1.47604200 -0.03028600  H -2.50167100 0.13300000 1.13896400 |
| anti-TS1 | junD | C 1.1662337258 0.0479993823 0.4294412766  O 0.8100715191 1.0402146330 -0.2811888344  O -0.5079354842 1.4181980597 -0.0263609101  H 0.5627167296 -0.1788476938 1.3122005281  O -0.4907115336 -1.1080047106 -0.5717934843  C -1.4102124454 -0.3161170901 -0.4483550245  F -2.1576860600 -0.3355784641 0.6925432559  H -1.9712388784 0.1598591407 -1.2608908112  C 2.4594993426 -0.6065296877 0.1253830194  H 3.1187844271 -0.5524670629 1.0040283436  H 2.2519048292 -1.6664121082 -0.0855449391  H 2.9352788282 -0.1405363984 -0.7453414200 |
|  | M11-L/MG3S | C 1.15475400 0.05950800 0.42626400  O 0.79866400 1.01912000 -0.27877900  O -0.47937400 1.38626100 -0.03429700  H 0.54035300 -0.18180100 1.30009400  O -0.47326900 -1.08953400 -0.56622400  C -1.37486900 -0.32076100 -0.42785600  F -2.08488400 -0.33963900 0.67374200  H -1.94052900 0.16266600 -1.24206600  C 2.41815700 -0.58986400 0.12580100  H 3.08816200 -0.55664000 0.99279600  H 2.21832700 -1.64828400 -0.08152600  H 2.90121300 -0.13925400 -0.74382800 |
| anti-TS2 | junD | C -1.3042870912 0.1480020784 -0.3378376574  O -0.6064315233 0.9704772366 0.3339188114  O 0.5288939892 1.3824743039 -0.3732182058  H -1.1412190343 0.1153127650 -1.4169469974  O 0.5131442368 -1.1664011014 -0.5654976751  C 1.4187175118 -0.3651273751 -0.3743875514  H 2.1346507798 -0.0046627481 -1.1237320082  F 1.9640586060 -0.2835562813 0.8726085354  C -2.4082381356 -0.5602784654 0.3497895974  H -3.3661903232 -0.3005658945 -0.1246109070  H -2.2394236912 -1.6394128424 0.2162206336  H -2.4241943249 -0.3143806757 1.4178884246 |
|  | M11-L/MG3S | C -1.29381000 0.15884500 -0.32899600  O -0.60251500 0.94792800 0.33494100  O 0.49978000 1.35113400 -0.34933300  H -1.11106800 0.11103700 -1.40703900  O 0.49916600 -1.15381100 -0.52044200  C 1.38799300 -0.37003900 -0.35545100  H 2.08188100 -0.00404600 -1.13343200  F 1.94592400 -0.27860200 0.82619800  C -2.37656200 -0.54277000 0.33645800  H -3.33736300 -0.30921000 -0.13713300  H -2.21273000 -1.61936500 0.20581800  H -2.41121500 -0.30922000 1.40260600 |
| syn-TS1 | junD | C 1.4416965667 0.0231169643 0.3494152855  O 1.0483672163 -1.1367155254 -0.0192856599  O -0.0730308992 -1.0931675944 -0.8416247655  H 2.1752799349 -0.0583070274 1.1603021494  O -0.5796649525 0.2102495050 1.2722748609  C -1.2825571068 -0.2334263609 0.3705241030  F -1.7977106145 0.6353303976 -0.5617528118  H -1.9316251307 -1.1149411483 0.4438827114  C 1.2898027599 1.2874994452 -0.4114227080  H 1.0925279236 2.1068883976 0.2911267084  H 2.2628669922 1.4735016336 -0.9002183478  H 0.4920403100 1.2201063131 -1.1551475254 |
|  | M11-L/MG3S | C 1.44425900 0.01350400 0.32788800  O 1.02126800 -1.11290000 -0.00245800  O -0.06686900 -1.05528200 -0.80311500  H 2.19138400 -0.06940700 1.12954200  O -0.58476400 0.23137000 1.24176300  C -1.25592000 -0.20819700 0.35158200  F -1.75330300 0.61539400 -0.55723200  H -1.90756500 -1.09795300 0.43132000  C 1.27185800 1.25757200 -0.39817900  H 1.06585900 2.07146300 0.30095800  H 2.23395800 1.48641500 -0.88432000  H 0.47782800 1.18815600 -1.13967500 |
| syn-TS2 | junD | C -1.3045162492 -0.1806935809 -0.6288280400  O -0.4922719953 -1.1419854702 -0.4060803166  O 0.1655736365 -1.0241276989 0.8183703104  H -1.6182395744 -0.1767337074 -1.6797052047  O 0.4183362814 1.2087227894 -0.3351094152  C 1.1833809013 0.5174794428 0.3341874972  H 1.4544036904 0.6978119881 1.3830776648  F 2.2057755687 -0.1230282370 -0.3051179906  C -2.0624024395 0.5840328827 0.3909161674  H -2.1279904400 1.6331460697 0.0771243634  H -3.0809220113 0.1569773847 0.4069968621  H -1.5986603687 0.5032261368 1.3769041019 |
|  | M11-L/MG3S | C -1.30994300 -0.20388400 -0.62026400  O -0.48078400 -1.11255200 -0.41687200  O 0.15683000 -0.98932700 0.77576900  H -1.64693300 -0.20975500 -1.66675100  O 0.40845600 1.20082700 -0.31585600  C 1.14738600 0.51002300 0.33257700  H 1.40767200 0.68226400 1.39533900  F 2.14648000 -0.09185700 -0.27444900  C -2.01860800 0.57134000 0.38000100  H -2.07370200 1.61656200 0.06694200  H -3.05051200 0.18780900 0.42007400  H -1.54387500 0.49337800 1.35622600 |
| anti-P1 | junD | C -0.9027251138 -0.1202079560 -0.3476597078  O -0.6272492229 1.2190206269 0.0011194921  O 0.8297795870 1.1966049665 0.1336720405  H -0.6140770896 -0.3046556875 -1.3973918456  O -0.0234906079 -0.8462813244 0.5122683663  C 1.1484137452 -0.1427606808 0.4498337666  F 1.9527547135 -0.6094553332 -0.5665578894  H 1.7173554108 -0.2171212267 1.3831128714  C -2.3410226540 -0.4364748792 -0.0453281301  H -2.9926494614 0.2006015161 -0.6584442774  H -2.5366898741 -1.4886271361 -0.2919004053  H -2.5430684328 -0.2611568856 1.0192287188 |
|  | M11-L/MG3S | C -0.89536700 -0.10727400 -0.35497100  O -0.59757500 1.17292000 0.03508000  O 0.80477800 1.12929600 0.00563500  H -0.65287500 -0.24378300 -1.43125100  O -0.01506600 -0.85879700 0.41786900  C 1.12113600 -0.14967600 0.42445700  F 1.99783300 -0.60791400 -0.45084800  H 1.59463600 -0.17287200 1.42273900  C -2.30476000 -0.42907100 -0.02853800  H -2.98183400 0.21519400 -0.59537700  H -2.51836000 -1.46724400 -0.29438700  H -2.48521500 -0.29129300 1.04154500 |
| anti-P2 | junD | C -1.0374328639 0.1687550702 -0.3925933118  O -0.3945528157 1.0776227052 0.4777077684  O 0.9151972306 1.1624639788 -0.1708809979  H -1.3202515844 0.6808369365 -1.3285289695  O -0.0192672926 -0.7919265335 -0.7021412886  C 1.1802837208 -0.1859293156 -0.3887225471  H 1.9285416149 -0.2614068224 -1.1871477631  F 1.6974738780 -0.7695610436 0.7388451646  C -2.1976491406 -0.4671416911 0.3253808007  H -2.9447288925 0.2996501111 0.5729127341  H -2.6586876314 -1.2146592222 -0.3342892225  H -1.8412152232 -0.9551371734 1.2418696327 |
|  | M11-L/MG3S | C -1.00585800 0.15536300 -0.36746800  O -0.38261600 1.03239200 0.48415500  O 0.87254300 1.12991700 -0.16346900  H -1.27762800 0.66825700 -1.31611500  O -0.02156600 -0.79184600 -0.63817600  C 1.15133200 -0.18315200 -0.37493300  H 1.87261600 -0.23831400 -1.20999100  F 1.69594300 -0.74072700 0.68994100  C -2.17623000 -0.45441000 0.31249800  H -2.92775900 0.30768100 0.53606400  H -2.63226800 -1.20236000 -0.34104300  H -1.86079800 -0.93923400 1.24094900 |
| syn-P1 | junD | C 1.1078722325 -0.0606064373 0.4493798745  O 0.8065395150 -1.2862272144 -0.1915540521  O -0.5516384142 -1.0365774275 -0.6654851468  H 1.8696099270 -0.2980499866 1.2042606967  O -0.1088804844 0.2247447012 1.1439364471  C -1.1137471310 -0.1691501688 0.2990150927  F -1.6321077800 0.8930279791 -0.4059763261  H -1.9433999140 -0.6273304773 0.8502920309  C 1.5244363519 1.0419026045 -0.5001589225  H 1.6625185372 1.9736812607 0.0661415811  H 2.4745797347 0.7636157564 -0.9773663267  H 0.7603114252 1.1996564100 -1.2718399487 |
|  | M11-L/MG3S | C 1.07652500 -0.05584500 0.44687700  O 0.75808300 -1.24541500 -0.16887200  O -0.50852000 -0.93327800 -0.69210300  H 1.83463000 -0.29776200 1.21074400  O -0.11107700 0.26610400 1.09713400  C -1.09315100 -0.15571600 0.28513700  F -1.67126000 0.84407400 -0.35618100  H -1.87711700 -0.68572300 0.85815900  C 1.52554400 1.01493000 -0.49287700  H 1.67351800 1.95299100 0.05004100  H 2.47180200 0.73247500 -0.96430300  H 0.77711700 1.18185200 -1.27311100 |
| syn-P2 | junD | C 0.9671915321 -0.1890776270 -0.5395325809  O 0.3349013512 1.0822047006 -0.5739330801  O -0.4887826752 1.0073576462 0.6359379426  H 1.1646727421 -0.4384440644 -1.5921279356  O -0.0444087333 -1.0698045647 -0.0332080709  C -1.0316774175 -0.2622304385 0.4920629949  H -1.3777195065 -0.6001791653 1.4762371287  F -2.1061889331 -0.2400310134 -0.3587905320  C 2.1991268407 -0.2311362674 0.3338265949  H 2.5883401836 -1.2585607293 0.3665426694  H 2.9678151412 0.4343405641 -0.0833678428  H 1.9475984747 0.0943189590 1.3523897118 |
|  | M11-L/MG3S | C 0.94385800 -0.18244300 -0.54493100  O 0.32239700 1.04673600 -0.58364300  O -0.44284600 0.97475000 0.61078900  H 1.17723800 -0.42836300 -1.59650200  O -0.04814000 -1.04356700 -0.08081600  C -0.99100100 -0.25726100 0.47489200  H -1.29360000 -0.60093900 1.47976800  F -2.05577300 -0.22966000 -0.30413400  C 2.14823100 -0.22767900 0.33658600  H 2.53968200 -1.24792100 0.39036200  H 2.93329500 0.42628600 -0.05468700  H 1.88752800 0.09881900 1.34835300 |

Table S14. Absolute energies in hartrees

| Species | Method | Energy (a.u.) |
| --- | --- | --- |
| FCHO | MW3X-L//D | -213.9470187 |
|  | W3X-L//D | -213.955548 |
|  | M11-L/MG3S | -213.757748 |
|  | MW2-F12//D | -213.9458461 |
|  | W2X//D | -213.9543754 |
|  | W2X//junD | -213.9541235 |
| CH_2_OO | MW3X-L//D | -189.714624 |
|  | W3X-L//D | -189.7225695 |
|  | M11-L/MG3S | -189.55266 |
|  | MW2-F12//D | -189.7116541 |
|  | W2X//D | -189.7195996 |
|  | W2X//junD | -189.7195993 |
| C1 | MW3X-L//D | -403.6741383 |
|  | W3X-L//D | -403.690813 |
|  | M11-L/MG3S | -403.320589 |
|  | MW2-F12//D | -403.6703989 |
|  | W2X//D | -403.6870743 |
|  | W2X//junD | -403.6864699 |
| C2 | MW3X-L//D | -403.6733737 |
|  | W3X-L//D | -403.6900454 |
|  | M11-L/MG3S | -403.319727 |
|  | MW2-F12//D | -403.6695996 |
|  | W2X//D | -403.6862713 |
|  | W2X//junD | -403.6856505 |
| TS1 | MW3X-L//D | -403.6705435 |
|  | W3X-L//D | -403.6872673 |
|  | M11-L/MG3S | -403.316603 |
|  | MW2-F12//D | -403.6672627 |
|  | W2X//D | -403.6839865 |
|  | W2X//junD | -403.6835141 |
| TS2 | MW3X-L//D | -403.6678238 |
|  | W3X-L//D | -403.6845351 |
|  | M11-L/MG3S | -403.313627 |
|  | MW2-F12//D | -403.6646344 |
|  | W2X//D | -403.6813457 |
|  | W2X//junD | -403.6808787 |
| P1 | MW3X-L//D | -403.7359573 |
|  | W3X-L//D | -403.7526299 |
|  | M11-L/MG3S | -403.37632 |
|  | MW2-F12//D | -403.7339807 |
|  | W2X//D | -403.7506533 |
|  | W2X//junD | -403.7502350 |
| P2 | MW3X-L//D | -403.738569 |
|  | W3X-L//D | -403.7552611 |
|  | M11-L/MG3S | -403.379725 |
|  | MW2-F12//D | -403.736605 |
|  | W2X//D | -403.7532971 |
|  | W2X//junD | -403.7528908 |
| anti-CH3CHOO | W3X-L//junD | -229.0662367 |
|  | W2X//junD | -229.0629202 |
|  | M11L | -228.859419 |
| syn-CH3CHOO | W3X-L//junD | -229.0719368 |
|  | W2X//junD | -229.0688182 |
|  | M11L | -228.86518 |
| anti-C1 | W3X-L//junD | -443.0370553 |
|  | W2X//junD | -443.0330725 |
|  | M11L | -442.711006 |
| anti-C2 | W3X-L//junD | -443.0360668 |
|  | W2X//junD | -443.0320502 |
|  | M11L | -442.70978 |
| syn-C1 | W3X-L//junD | -443.0393362 |
|  | W2X//junD | -443.0353167 |
|  | M11L | -442.713306 |
| syn-C2 | W3X-L//junD | -443.0385853 |
|  | W2X//junD | -443.0345358 |
|  | M11L | -442.712574 |
| anti-TS1 | W3X-L//junD | -443.0354134 |
|  | W2X//junD | -443.0318443 |
|  | M11L | -442.709256 |
| anti-TS2 | W3X-L//junD | -443.0330693 |
|  | W2X//junD | -443.0295854 |
|  | M11L | -442.706836 |
| syn-TS1 | W3X-L//junD | -443.0313219 |
|  | W2X//junD | -443.027889 |
|  | M11L | -442.705659 |
| syn-TS2 | W3X-L//junD | -443.0284046 |
|  | W2X//junD | -443.0250121 |
|  | M11L | -442.703116 |
| anti-P1 | W3X-L//junD | -443.0960068 |
|  | W2X//junD | -443.0937171 |
|  | M11L | -442.767317 |
| anti-P2 | W3X-L//junD | -443.0985224 |
|  | W2X//junD | -443.0962458 |
|  | M11L | -442.770487 |
| syn-P1 | W3X-L//junD | -443.0931434 |
|  | W2X//junD | -443.0908429 |
|  | M11L | -442.764427 |
| syn-P2 | W3X-L//junD | -443.0968098 |
|  | W2X//junD | -443.094528 |
|  | M11L | -442.768434 |

**Figures**


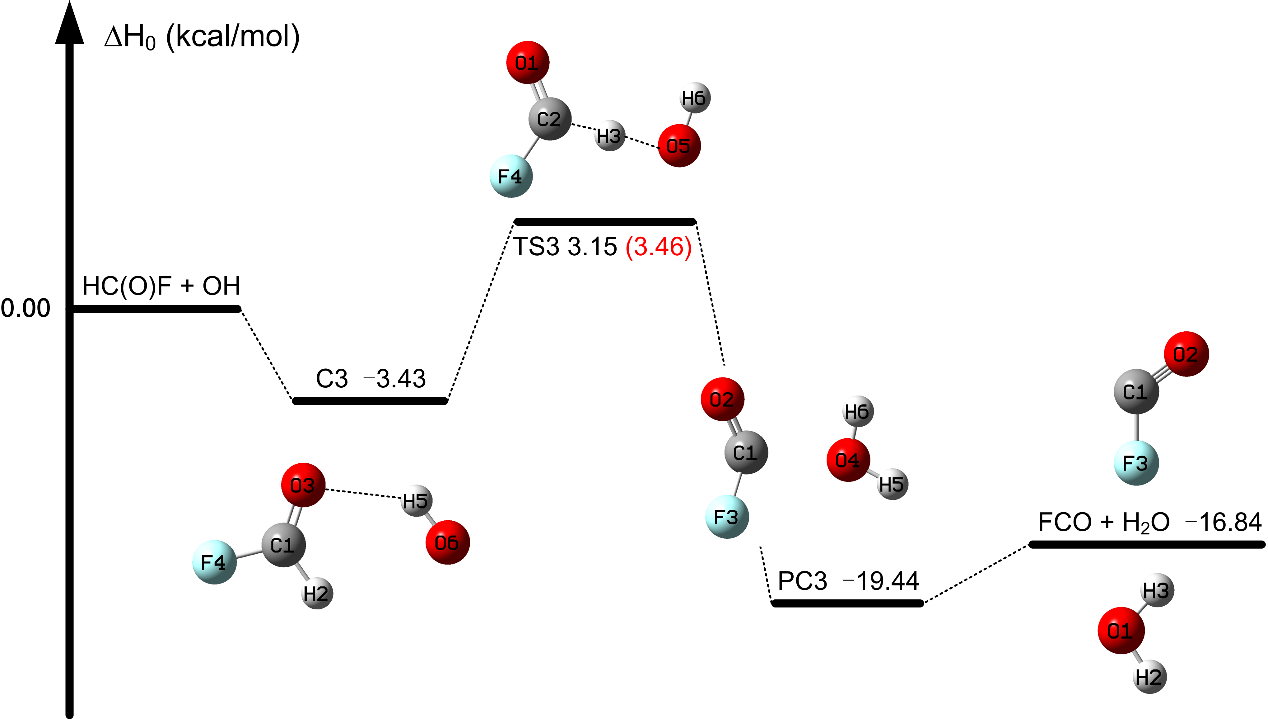


Figure S1. Enthalpies for the FCHO + OH reaction at 0 K.

In black: Enthalpy profile calculated by M08-HX/MG3S.

In red: Enthalpy of activation calculated by W3X-L//CCSD(T)-F12b/jun-cc-pVDZ.


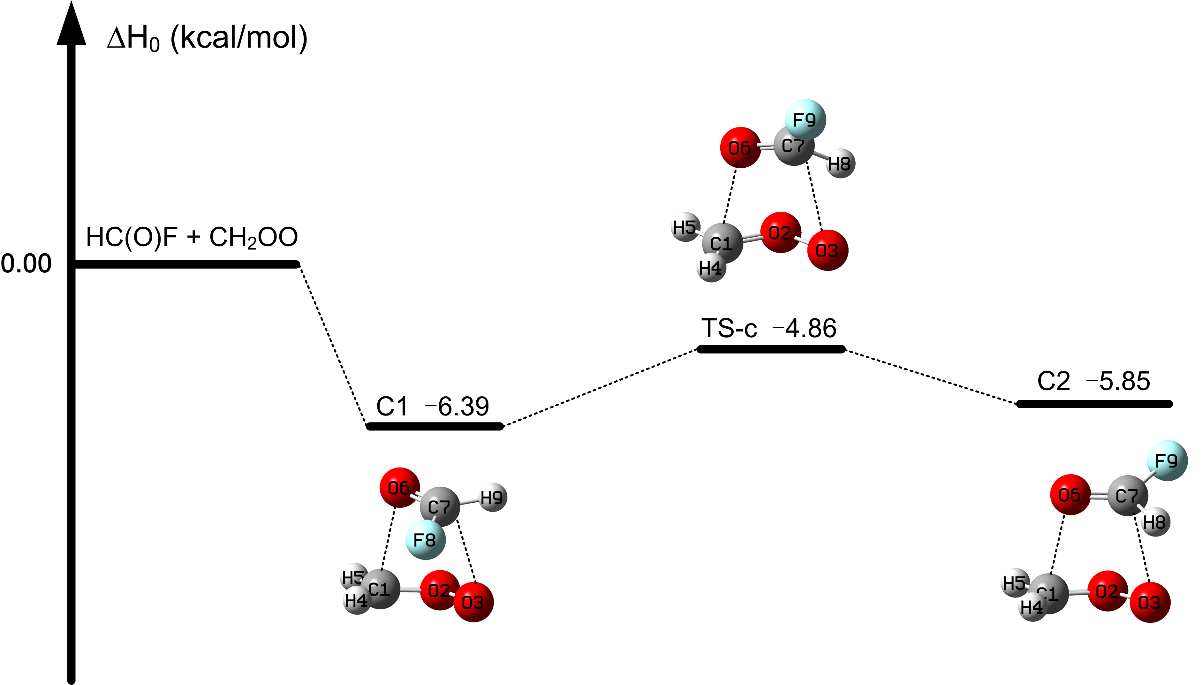


Figure S2. Enthalpy profile at 0 K for the conversion of C1 to C2 as calculated by M11-L/MG3S.

**References**

[1]. M. J. Frisch, G. W. Trucks, H. B. Schlegel, et al. *Gaussian 16 Rev. C.01*, Wallingford, CT, 2016.

[2]. Y. Zhao, R. Peverati, K. Tang, S. Luo, H. Yu, X. He, D. Truhlar, MN-GFM 6.7; Department of Chemistry. University of Minnesota: Minneapolis, MN 55455-0431 (2015).

[3]. H.-J. K. Werner, P. J.; Knizia, G.; Manby, F. R.; Schütz, M.; Celani, P.; Györffy, W.; Kats, D.; Korona, T.; Lindh, R.; Mitrushenkov, A.; Rauhut, G.; Shamasundar, K. R.; Adler, T. B.; Amos, R. D.; Bennie, S. J.; Bernhardsson, A.; Berning, A.; Cooper, D. L.; Deegan, M. J. O.; Dobbyn, A. J.; Eckert, F.; Goll, E.; Hampel, C.; Hesselmann, A.; Hetzer, G.; Hrenar, T.; Jansen, G.; Köppl, C.; Lee, S. J. R.; Liu, Y.; Lloyd, A. W.; Ma, Q.; Mata, R. A.; May, A. J.; McNicholas, S. J.; Meyer, W.; Miller, T. F.; Mura, M. E.; Nicklaß, A.; O'Neill, D. P.; Palmieri, P.; Peng, D.; Pflüger, K.; Pitzer, R.; Reiher, M.; Shiozaki, T.; Stoll, H.; Stone, A. J.; Tarroni, R.; Thorsteinsson, T.; Wang, M.; Welborn, M, Molpro: a general‐purpose quantum chemistry program package. WIREs Comput. Mol. Sci. 2 (2012) 242-253.

[4]. M. Kállay, P. R. Nagy, D. Mester, et al., The MRCC program system: Accurate quantum chemistry from water to proteins. J. Chem. Phys. 152 (2020) 074107.

[5]. M. N. Kállay, P. R.; Mester, D.; Rolik, Z.; Samu, G.; Csontos, J.; Csóka, J.; Szabó, P. B.; Gyevi-Nagy, L.; Hégely, B.; Ladjánszki, I.; Szegedy, L.; Ladóczki, B.; Petrov, K.; Farkas, M.; Mezei, P. D.; Ganyecz, Á, MRCC, a string-based quantum chemical program suite. <www.mrcc.hu>.

[6]. J. Zheng, J. Bao, R. Meana-Pañeda, et al., Polyrate, version 2017-C. University of Minnesota, Minneapolis, MN (2018).

[7]. J. Zheng, L. Bao, S. Zhang, J. Corchado, Y. Chuang, E. Coitiño, B. Ellingson, D. Truhlar, Gaussrate, version 2017-B. University of Minnesota: Minneapolis (2018).

[8]. J. L. Bao, D. G. Truhlar, Variational transition state theory: theoretical framework and recent developments. Chem. Soc. Rev. 46 (2017) 7548-7596.

[9]. Y. Georgievskii, S. J. Klippenstein, Variable reaction coordinate transition state theory: Analytic results and application to the C_2_H_3_ + H→ C_2_H_4_ reaction. J. Chem. Phys. 118 (2003) 5442-5455.

[10]. J. Zheng, S. Zhang, D. G. Truhlar, Density Functional Study of Methyl Radical Association Kinetics. J. Phys. Chem. A 112 (2008) 11509-11513.

[11]. J. L. Bao, X. Zhang, D. G. Truhlar, Barrierless association of CF2 and dissociation of C2F4 by variational transition-state theory and system-specific quantum Rice–Ramsperger–Kassel theory. Proc. Natl. Acad. Sci. USA. 113 (2016) 13606-13611.

[12]. B. Long, Y. Wang, Y. Xia, X. He, J. L. Bao, D. G. Truhlar, Atmospheric Kinetics: Bimolecular Reactions of Carbonyl Oxide by a Triple-Level Strategy. J Am Chem Soc. 143 (2021) 8402-8413.

[13]. B. Long, J. L. Bao, D. G. Truhlar, Unimolecular reaction of acetone oxide and its reaction with water in the atmosphere. Proc. Natl. Acad. Sci. USA. 115 (2018) 6135-6140.

[14]. B. Long, J. L. Bao, D. G. Truhlar, Atmospheric Chemistry of Criegee Intermediates: Unimolecular Reactions and Reactions with Water. J. Am. Chem. Soc. 138 (2016) 14409-14422.

[15]. B. Long, J. L. Bao, D. G. Truhlar, Kinetics of the Strongly Correlated CH3O + O2 Reaction: The Importance of Quadruple Excitations in Atmospheric and Combustion Chemistry. J. Am. Chem. Soc. 141 (2019) 611-617.

[16]. X.-F. Tan, B. Long, D.-S. Ren, W.-J. Zhang, Z.-W. Long, E. Mitchell, Atmospheric chemistry of CH_3_CHO: the hydrolysis of CH_3_CHO catalyzed by H_2_SO4. Phys. Chem. Chem. Phys. 20 (2018) 7701-7709.

[17]. X.-F. Tan, L. Zhang, B. Long, New mechanistic pathways for the formation of organosulfates catalyzed by ammonia and carbinolamine formation catalyzed by sulfuric acid in the atmosphere. Phys. Chem. Chem. Phys. 22 (2020) 8800-8807.

[18]. B. Long, Y. Wang, Y. Xia, X. He, J. L. Bao, D. G. Truhlar, Atmospheric Kinetics: Bimolecular Reactions of Carbonyl Oxide by a Triple-Level Strategy. J. Am. Chem. Soc. 143 (2021) 8402-8413.

[19]. B. Chan, L. Radom, W3X: A Cost-Effective Post-CCSD(T) Composite Procedure. J. Chem. Theory Comput. 9 (2013) 4769-4778.

[20]. G. Knizia, T. B. Adler, H.-J. Werner, Simplified CCSD(T)-F12 methods: Theory and benchmarks. J. Chem. Phys. 130 (2009) 054104.

[21]. E. Papajak, J. Zheng, X. Xu, H. R. Leverentz, D. G. Truhlar, Perspectives on Basis Sets Beautiful: Seasonal Plantings of Diffuse Basis Functions. J. Chem. Theory Comput. 7 (2011) 3027-3034.

[22]. Y. Zhao, D. G. Truhlar, Exploring the Limit of Accuracy of the Global Hybrid Meta Density Functional for Main-Group Thermochemistry, Kinetics, and Noncovalent Interactions. J. Chem. Theory Comput. 4 (2008) 1849-1868.

[23]. B. J. Lynch, Y. Zhao, D. G. Truhlar, Effectiveness of Diffuse Basis Functions for Calculating Relative Energies by Density Functional Theory. J. Phys. Chem. A 107 (2003) 1384-1388.

[24]. R. Peverati, D. G. Truhlar, M11-L: A Local Density Functional That Provides Improved Accuracy for Electronic Structure Calculations in Chemistry and Physics. J. Phys. Chem. Lett. 3 (2012) 117-124.

[25]. K. Raghavachari, G. W. Trucks, J. A. Pople, M. Head-Gordon, A fifth-order perturbation comparison of electron correlation theories. Chem. Phys. Lett. 157 (1989) 479-483.

[26]. Y. J. Bomble, J. F. Stanton, M. Kállay, J. Gauss, Coupled-cluster methods including noniterative corrections for quadruple excitations. J. Chem. Phys. 123 (2005) 054101.

[27]. B. Chan, L. Radom, W2X and W3X-L: Cost-Effective Approximations to W2 and W4 with kJ mol^–1^ Accuracy. J. Chem. Theory Comput. 11 (2015) 2109-2119.

[28]. N. Sylvetsky, K. A. Peterson, A. Karton, J. M. Martin, Toward a W4-F12 approach: Can explicitly correlated and orbital-based ab initio CCSD (T) limits be reconciled? J. Chem. Phys. 144 (2016) 214101.

[29]. K. A. Peterson, T. B. Adler, H.-J. Werner, Systematically convergent basis sets for explicitly correlated wavefunctions: The atoms H, He, B–Ne, and Al–Ar. J. Chem. Phys. 128 (2008) 084102.

[30]. A. Halkier, T. Helgaker, P. Jørgensen, W. Klopper, H. Koch, J. Olsen, A. K. Wilson, Basis-set convergence in correlated calculations on Ne, N_2_, and H_2_O. Chem. Phys. Lett. 286 (1998) 243-252.

[31]. D. G. Truhlar, B. C. Garrett, Variational transition-state theory. Acc. Chem. Res. 13 (1980) 440-448.

[32]. Y. P. Liu, G. C. Lynch, T. N. Truong, D. H. Lu, D. G. Truhlar, B. C. Garrett, Molecular modeling of the kinetic isotope effect for the [1,5]-sigmatropic rearrangement of cis-1,3-pentadiene. J. Am. Chem. Soc. 115 (1993) 2408-2415.

[33]. I. Alecu, J. Zheng, Y. Zhao, D. G. Truhlar, Computational thermochemistry: scale factor databases and scale factors for vibrational frequencies obtained from electronic model chemistries. J Chem Theory Comput. 6 (2010) 2872-2887.

1. * Corresponding author email addresses:

   [wwwltcommon@sina.com](mailto:wwwltcommon@sina.com) (Bo Long)

   [truhlar@umn.edu](mailto:truhlar@umn.edu) (Donald G. Truhlar) [↑](#footnote-ref-1)
